# Pathogenic signal peptide variants in the human genome

**DOI:** 10.1093/nargab/lqad093

**Published:** 2023-10-18

**Authors:** Sneider Alexander Gutierrez Guarnizo, Morgana K Kellogg, Sarah C Miller, Elena B Tikhonova, Zemfira N Karamysheva, Andrey L Karamyshev

**Affiliations:** Department of Cell Biology and Biochemistry, Texas Tech University Health Sciences Center, Lubbock, TX 79430, USA; Department of Cell Biology and Biochemistry, Texas Tech University Health Sciences Center, Lubbock, TX 79430, USA; Department of Cell Biology and Biochemistry, Texas Tech University Health Sciences Center, Lubbock, TX 79430, USA; Department of Cell Biology and Biochemistry, Texas Tech University Health Sciences Center, Lubbock, TX 79430, USA; Department of Biological Sciences, Texas Tech University, Lubbock, TX 79409, USA; Department of Cell Biology and Biochemistry, Texas Tech University Health Sciences Center, Lubbock, TX 79430, USA

## Abstract

Secreted and membrane proteins represent a third of all cellular proteins and contain N-terminal signal peptides that are required for protein targeting to endoplasmic reticulum (ER). Mutations in signal peptides affect protein targeting, translocation, processing, and stability, and are associated with human diseases. However, only a few of them have been identified or characterized. In this report, we identified pathogenic signal peptide variants across the human genome using bioinformatic analyses and predicted the molecular mechanisms of their pathology. We recovered more than 65 thousand signal peptide mutations, over 11 thousand we classified as pathogenic, and proposed framework for distinction of their molecular mechanisms. The pathogenic mutations affect over 3.3 thousand genes coding for secreted and membrane proteins. Most pathogenic mutations alter the signal peptide hydrophobic core, a critical recognition region for the signal recognition particle, potentially activating the Regulation of Aberrant Protein Production (RAPP) quality control and specific mRNA degradation. The remaining pathogenic variants (about 25%) alter either the N-terminal region or signal peptidase processing site that can result in translocation deficiencies at the ER membrane or inhibit protein processing. This work provides a conceptual framework for the identification of mutations across the genome and their connection with human disease.

## Introduction

Eukaryotic cells have multiple intracellular compartments that require coordinated protein sorting. Ribosomes synthesize thousands of proteins that must be transported to different organelles, integrated into membranes, or secreted outside of the cell (1). Different intrinsic signals in the amino acid sequence act like postal codes to deliver proteins to specific cellular locations (2,3). The most numerous targeting signals include signal peptides, N-terminal amino acid sequences that direct the targeting and translocation of many secreted and membrane proteins to the endoplasmic reticulum (ER) (4–7). Secreted and membrane proteins represent over 30% of the human proteome and participate in essential biological processes such as cell signaling, transport, and cell recognition (8–10). Defects in the trafficking of secreted or membrane proteins contribute to the pathogenesis of many human diseases (7,11–15).

**Figure 1.**
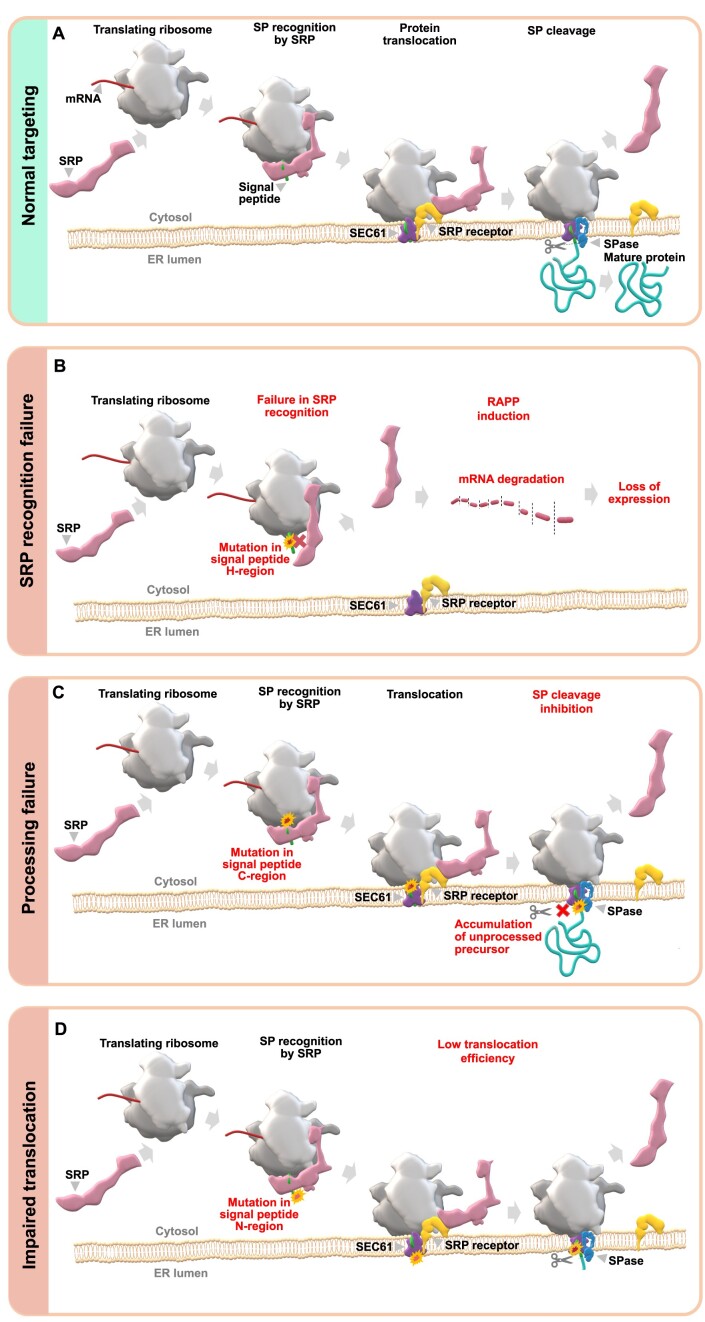
Mutations in signal peptides affect different molecular mechanisms. (**A**) Illustration of a normal targeting of secreted/membrane proteins to ER. When a nascent polypeptide containing signal peptide is exposed from the ribosome tunnel, it is co-translationally recognized by SRP. Then, the SRP-ribosome nascent chain complex is targeted to SRP receptor in the ER membrane. Finally, the nascent polypeptide is co-translationally translocated into the ER lumen through protein-conducting channel in SEC61 translocon, the signal peptide is cleaved off by signal peptidase (SPase), and the mature protein is released from the ER membrane and transported further to extracellular space, or integrated into the plasma membrane, or remained in the ER lumen. (**B**) Mutations affecting hydrophobicity of signal peptide H-region lead to SRP recognition failure, activation of the RAPP pathway and degradation of the protein's mRNA. (**C**) Mutations in signal peptide cleavage site may affect protein processing leading to accumulation of unprocessed protein in ER. (**D**) Mutations in signal peptide N-terminus may affect its interaction with SEC61 translocon decreasing protein translocation efficiency.

Co-translational protein targeting involves nascent peptide recognition by a ribonucleoprotein complex known as the signal recognition particle (SRP). SRP co-translationally binds the signal peptides or transmembrane domains of secreted or membrane proteins once they are exposed from the ribosome's exit tunnel, forming SRP-ribosome-nascent-chain complexes (SRP-RNC). When SRP binds to the RNC, it temporarily arrests translation to allow targeting of the complex to the SRP receptor in the ER membrane. SRP then hands the RNC to the SEC61 translocon for co-translational translocation into the ER lumen. Signal peptidase cleaves signal peptides after translocation, releasing the protein into the ER lumen. These events are summarized in Figure 1A and have been described in detail in multiple reviews (7,16–21).

**Figure 2.**
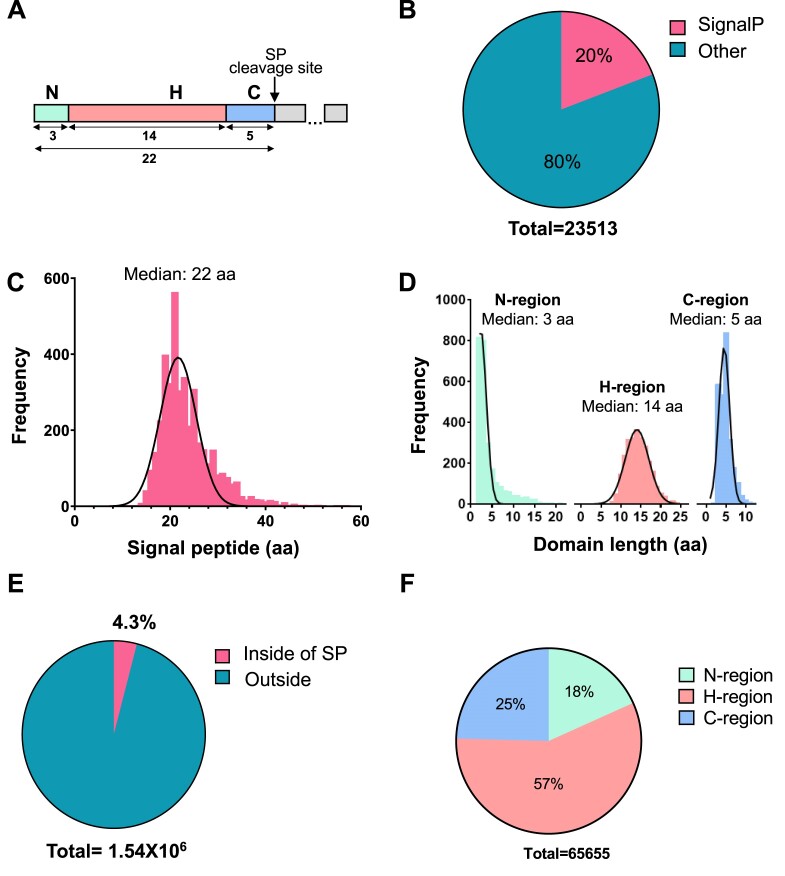
Detection of signal peptides and signal peptide missense variants at the whole human genome. (**A**) Graphical representation of signal peptide regions: N-terminal region (N), hydrophobic region (H), C-terminal region with cleavage site for signal peptidase (C). The numbers are median lengths of the human signal peptides and their regions as determined in this work as shown in the figure panels C and D. (**B**) Relative number of proteins containing cleavable signal peptides among other proteins in human proteome. Genes coding proteins containing signal peptide sequences were selected from the UniProtKB/Swiss-Prot and verified by SignalP 6.0. The number of proteins were determined by the exclusive stable Ensembl peptide identifiers. (**C**) Distribution of signal peptides relatively to their length in amino residues among all signal peptides. (**D**) Frequency distribution of signal peptide regions per amino acid sequence length in all signal peptides. (**E**) Percentage of missense variants detected inside and outside of the signal peptide coding sequences. (**F**) Distribution of the detected signal peptide missense variants between different signal peptide regions. The signal peptide variants include those located in the position +1 because it may affect signal peptide cleavage (protein processing).

Signal peptides do not have a consensus sequence, but contain three distinct regions with conserved physicochemical features: a positively charged N-terminal region (N-region); a hydrophobic region (H-region), which consists of primarily aliphatic amino acid residues; and a C-terminal region (C-region), which includes the cleavage site recognized by signal peptidase (4,5,22) (Figure 2A). The H-region is the most critical region for SRP recognition, which is directly involved in hydrophobic interactions between the signal peptide and the methionine-lined binding pocket of the SRP54 subunit. Some mutations in the H-region have been shown to lead to severe defects in SRP recognition of the signal peptide (15,23–25) (Figure 1B). In contrast, some signal peptide mutations in the C-region have been shown to disrupt signal peptide cleavage, leading to the accumulation of precursor protein in the ER membrane (26,27) (Figure 1C). Mutations in the N-region can interfere with protein translocation efficiency, induce protein mistargeting and aggregation, or can lead to the accumulation of precursor proteins at the ER membrane because the signal peptide is not oriented correctly in the translocon (14,23,28,29) (Figure 1D).

Misfolded and mistargeted proteins are often cytotoxic. Cells have developed several quality control mechanisms to prevent mistargeting or the accumulation of misfolded secretory and membrane proteins (30–32). These quality control mechanisms occur in the cytosol during protein targeting or at the ER during translocation. One of them is the preemptive quality control pathway, the Regulation of Aberrant Protein Production (RAPP), which senses the failure of SRP to recognize signal peptides, and specifically targets secretory and membrane protein mRNAs for degradation (15,24,25,33,34) (Figure 1B). Other quality control mechanisms in the cytosol involve the recognition of aberrant proteins that have already been synthesized and released from the ribosome. These are known to be targeted for degradation by the ubiquitin/proteasome system in the cytoplasm (35). At the ER, misfolded or accumulated proteins activate the Unfolded Protein Response (UPR), or ER-associated degradation (ERAD) (32,36–38). Unprocessed precursors due to mutations at the signal peptidase cleavage site or in the N-region are modified with ubiquitin and subjected to degradation by the proteasome, reviewed in (39).

The association between signal peptide variants and human diseases is likely underestimated. While there are several reports of missense variants in signal peptides that are associated with human diseases, researchers have experimentally validated only a few variants (7,15,24,40–55). Since it is technically impossible to study the contribution of all signal peptide variants to the pathogenesis of human diseases, strategies of classification and prediction are needed to identify pathogenic signal peptide variants. Here, we report single nucleotide polymorphisms (SNPs) in signal peptide coding sequences across the human genome and predicted their pathogenicity. Using a rational stepwise classification strategy, we identified more than eleven thousand predicted pathogenic variants (PPVs) and their possible molecular mechanisms in a number of human diseases. We validated some PPVs by computational modeling of their interactions with SRP compared to the wildtype sequence. Our analysis provides a conceptual framework for how PPVs may alter the targeting, translocation, and processing of more than three thousand membrane and secreted proteins. Identifying PPVs in signal peptides will contribute to our understanding of the secretory pathway and the genetic basis for human diseases.

## Materials and methods

### Identification of human proteins with signal peptides

Gene-coding proteins with signal peptides were identified by using the UniProtKB/Swiss-Prot database. The search comprised the following parameters: ‘annotation:(type: signal) AND reviewed: yes AND organism: ‘Homo sapiens (Human) [9606]’ (56). These proteins were annotated with stable ensemble protein IDs (https://m.ensembl.org/info/genome/stable_ids/index.html) and the amino acid sequences were recovered as .fasta format by using the function ‘getSequence’ of the BiomaRt package (57). The SignalP 6.0 slow mode algorithm through Python Programming Language 3.10.0 was used to predict the presence of signal peptides and signal peptide regions (https://www.python.org/) (58). See Supplementary File S1.

### Identification of human signal peptide variants

To identify genetic variants associated with signal peptides, the single nucleotide variation database (dbSNP) from NCBI was used (59). The SnpEff algorithm predicted each variant's effect at the protein level, using the GRCh38.p13 human genome for reference (60), Ensembl's Variant Effect Predictor algorithm selected missense variants that affected canonical proteins (61).

The generated datasets containing (i) signal peptide proteins and (ii) human missense variants were matched based on the Ensembl protein IDs. The variants that fell within the signal peptide protein sequence were selected. The last amino acid residue in the signal peptide just before the cleavage site was denoted as ‘–1’, and the first amino acid residue in the mature part as ‘+1’ (the location of the signal peptide cleavage site is between the –1 and + 1 amino acid positions). The +1 amino acid residues were included in the analysis of wild type signal peptides. The mutant versions were built by replacing the reference (wildtype) amino acid with the respective amino acid incorporated by the missense variant in Microsoft Excel.

### Identification of signal peptide pathogenic variants

The identification of a predicted pathogenic variant (PPV) included a stepwise strategy of classification performed partially in the R language and with Microsoft Excel (Supplementary Files S3, S9). Three parameters were defined to detect missense variants that interfere with signal peptide recognition in the hydrophobic core:

- missense variants that modify the hydrophobic core (H-region);
- variants that decrease the hydrophobicity;
- variants that reduce the potential binding between the signal peptide and SRP.

First, missense variants outside of the H-region were filtered out. Next, the change in hydrophobicity was estimated by subtracting the hydrophobicity of the amino acid of the variant from the amino acid of the wild type. The hydrophobicity estimation was based on the Kyte & Doolittle scale (62). Signal peptides with significant hydrophobicity decreases were considered for further analysis of the potential change in SRP interaction via the Boman Index. The potential protein interaction index, or the Boman Index, was used to measure the variant's overall impact on the signal peptide interaction with SRP. This parameter was estimated by subtracting the Boman Index of the wild type amino acid from its variant, followed by multiplication by 100 for scaling (63). Based on previous experiments (15), the change in the Boman Index ≤–20 was used as the cutoff for possible SRP recognition failure, RAPP activation, and induced mRNA decay. The variants that fulfilled these three parameters were classified as PPVs in the H-region.

Detection of variants leading to signal peptidase failure was based on the selection of missense variants modifying the signal peptide C-region or the position + 1. The amino acid position ‘+1’, ‘–1’ and ‘–3’ were identified based on the cleavage site. The predicted variants incorporating less frequent amino acids and potentially affecting signal peptidase recognition were classified as PPVs.

To identify pathogenic variants in the N-region, the missense variants that incorporate negatively charged amino acids aspartate (D) and glutamate (E) instead of polar, non-polar, or positively charged amino acids were also classified as PPVs.

### Computational modelling of the signal recognition particle targeting a signal peptide

DeepMind's AlphaFold (64,65) was used to predict how SRP54 and the signal peptide dock *de novo*. AlphaFold uses machine learning and artificial intelligence to predict secondary, tertiary, and quaternary structures from the primary amino acid sequence. AlphaFold minimizes the root mean square deviation (RMSD) between atoms. RMSD is ultimately a measure of accuracy, the square root of the differences between predicted and observed values. RMSD values closer to or below 0 indicate better models than those above 0. Thus, minimizing RMSD leads to more optimal models. Rosetta Online Server that Includes Everyone (ROSIE) uses AlphaFold's model as a starting point before predicting how SRP54 and the signal peptide interact by further minimization of the RMSD and its own internal Rosetta Total Score generated by 1000 model iterations (66,67).

Models are submitted to ModelArchive (modelarchive.org). The individual links for the models are:

SRP54 with ALK WT signal peptide: modelarchive.org/doi/10.5452/ma-owxf7

SRP54 with ALK W8R signal peptide: modelarchive.org/doi/10.5452/ma-w701z

SRP54 with ALK S15Y signal peptide: modelarchive.org/doi/10.5452/ma-cm6gn

The models were further validated according to CAPRI quality assessment criteria with correct models defined as follows:

Fnat (frequency of native contacts) > 0.1 -OR-

Ligand rms (root mean squared) < 10.0 -AND- interface rms (irms) < 4.0

Supplementary File S11 provides validation of SRP54 Rosetta models with different ALK signal peptides. SRP54 with ALK WT and SRP54 with ALK S15Y are valid as models, but SRP54 with ALK W8R is not valid as a model that demonstrates SRP54 and ALKW8R do not interact, and, thus, in agreement with our prediction.

### Molecular images

The molecular images were created in PyMol (68). The SRP subunits were aligned on 7SL RNA using PDB coordinates 1RY1, 5WRW, 5WRV, 4P3F and 1MFQ (69–72). The image of the human signal peptidase complex was prepared by using coordinates 7P2P (73).

### Association of signal peptide variants and human diseases

Genes with a PPV were surveyed for their association with human disease. The analysis was based on the Genetic Association Database (GAD) using the DAVID Bioinformatics Resources 6.8, NIAID/NIH. We filtered significantly enriched diseases and disease categories by a false discovery rate lower than 0.05. We estimated the association between a decrease in protein expression and disease or cell phenotype using the function ‘BioProfiler’ in IPA software (Qiagen, Version: 763620684).

### Association of signal peptide genes and biological processes

The set of genes with PPVs was analyzed based on Protein ANalysis THrough Evolutionary Relationships, a bioinformatical analysis tool that groups genes based on evolutionary relationships (74) (Supplementary File S10). We used a cut-off of 50 genes to define a group of genes that clustered for a biological process.

## Results

### Signal peptides in human proteome

There were 3607 genes encoding proteins with annotated signal peptides in the UniProtKB/Swiss-Prot database (56) (Supplementary File S1 UniProt). We assigned 4504 different proteins stable Ensembl peptide identifiers to avoid redundancy (see Methods) and to accurately denote different isoforms (Supplementary File S1 Ensembl). To validate the presence of signal peptides in the recovered amino acid sequences and to determine the signal peptide regions, we analyzed these by the predictive algorithm SignalP6.0 that was recently published using the slow mode (58). As a result, we confirmed the presence of 4142 different proteins with signal peptides (Supplementary File S1 SignalP6.0Slow). We chose the most abundant protein isoforms as the reference for canonical transcripts. Proteins with cleavable signal peptides contribute to 20% of the total human proteome (Figure 2B, Supplementary File S1). Signal peptides are variable in length with a median of 22 amino acids (Figure 2A, C). As previously mentioned, signal peptides have conserved functional domains. To annotate these domains for each signal peptide, we used the slow mode algorithm of SignalP6.0, which defines the borders of each of the three domains for each signal peptide (58). These domains vary in length with median values of 3 residues for the N-region, 14 residues for the H-region and 5 residues in the C-region (Figure 2A, D, Supplementary File S1). The C-region is the most conserved in length. Furthermore, signal peptide regions are characterized by specific features. The N-region contains 16.5% of positively charged amino acids, with almost three times more arginine than lysine. Other amino acids such as alanine, glycine, and proline are also frequently observed in the N-terminal region with a frequency of 9.78%, 8.24% and 9.22%, respectively. The least frequent amino acid in the N-region is tyrosine (0.28%). The central H-domain predominantly contains hydrophobic amino acids such as leucine (36.93%), valine (8.8%), and alanine (9.68%), while aspartate (0.21%), asparagine (0.39%), and lysine (0.41%) are rare. Finally, the C-region contains mostly alanine (20.14%), glycine (16.16%), and serine (11.06%), while the presence of methionine (0.9%), phenylalanine (0.99%), and asparagine (1%) are rare (Supplementary File S2). Overall, these results highlight that although human SPs are variable in amino acid sequences, signal peptide regions preferentially contain specific groups of amino acids that support conserved functional features.

### Missense variants modifying signal peptides

To detect signal peptide variants, we used the dbSNP-NCBI repository to recover all reported human single nucleotide polymorphisms (SNPs) (75). We further categorized these SNPs by effect: synonymous, missense, upstream, downstream, in-frame and out-of-frame shifts, and deletions using SnpEff 5.0 software (60). We selected missense variants and annotated these SNPs with the Variant Effect Predictor algorithm (61). Out of 1 540 002 missense variants in genes coding for proteins with signal peptides, 65 655 or over 4% of missense variants were actually located within the signal peptides of 3506 unique proteins (Figure 2E, Supplementary File S3). This means that 82% of signal peptide-containing proteins have at least one missense mutation in their signal peptide (Supplementary File S3). The distribution of missense variants showed that 12 003 change the N-region; 37 526 change the H-region; and 16 126 change the C-region. (Figure 2F, Supplementary File S3). Most variants (57%) were found in the H-region, the lengthiest domain in signal peptides.

As stated above, we used two approaches for detection of signal peptides in the proteins, UniProt and SignalP6.0 (slow mode). Comparing signal peptides detected by these methods, we observed that while the majority (83%) of the signal peptides are the same regardless of the method used, 17% of signal peptides have alternative lengths (see Supplementary Text andSupplementary Figure S1). Moreover, we observed that UniProt and SignalP6.0 distributed residues differently between the H- and C-regions. Although it is impossible to evaluate whether UniProt or SignalP6.0 is more precise without reliable experimental data, our results suggest that the research groups working with secretory proteins need to pay careful attention to the algorithms used to predict signal peptides. Although we completed analysis of all alternative variants (see Supplementary Text, Supplementary Files andSupplementary Figures S1–S3), we chose to utilize SignalP6.0 exclusively as it is the most up-to-date software for signal peptide prediction.

### Numerous variants modifying the signal peptide H-region are predicted to disrupt SRP recognition and activate RAPP

**Figure 3.**
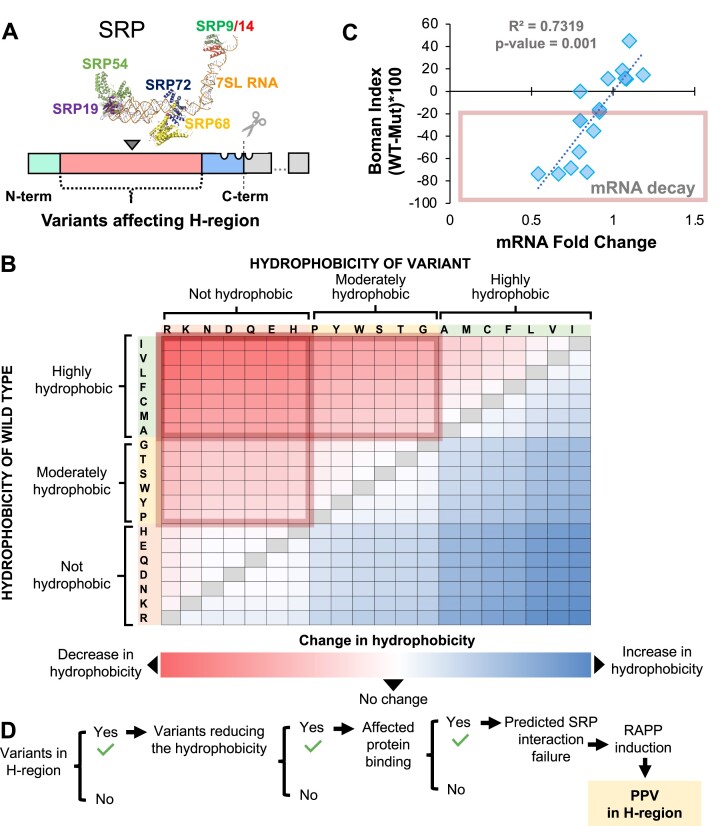
Gene variants affecting the signal peptide H-region can be pathogenic by inducing SRP recognition failure. (**A**) Schematic representation of positions of missense variants modifying the signal peptide H-region sequence. These variants can affect interaction with SRP. SRP image was created in PyMol by aligning the SRP subunits on 7SL RNA. The SRP subunits are marked. (**B**) Hydrophobicity scale of amino acids substitutions resulted from missense variants relatively to wildtype amino acids in signal peptides. Color gradient represents the effect on the hydrophobicity after replacing wildtype amino acid with a mutant variant—blue is high and red is low hydrophobicity. The scores of hydrophobicity per amino acid were estimated by Kyte-Doolittle scale. (**C**) Missense variants potentially reducing the SRP interaction and activating protein's mRNA degradation via RAPP pathway. The changes in the potential protein interaction across signal peptide sequences (Boman index, Y-axis) positively correlate with the changes in protein's mRNA levels experimentally detected (X-axis). Correlation analysis was completed by Pearson test. *R*-squared: 0.732. *F* distribution value: 40.95. Freedom degrees of numerator: 1. Freedom degrees of denominator: 15. *P* value: <0.0001. (**D**) Summary of the strategy used for the identification of PPVs affecting signal peptide H-region.

The central hydrophobic signal peptide H-region contains most of the missense variants (*n*: 37 526, or 57%) (Figure 2F). This region is crucial for signal peptide interaction with SRP, the first step for targeting secreted proteins to the ER (23) (Figure 1A). Previously, we have shown that mutations that reduce the hydrophobicity of the H-region impair SRP recognition and lead to mutant protein mRNA degradation through the RAPP pathway (15,23–25) (Figure 1B). To predict mutations that activate RAPP, we selected missense variants in the H-region protein coding sequence (Figure 3A) and classified these variants by their effect on H-region hydrophobicity. SRP recognition of the H-region is impaired when changes between wildtype and mutant signal peptides result in a notable hydrophobicity decrease (Figure 3B). In contrast, SRP recognition still occurs when hydrophobicity increased or slightly changed. We previously demonstrated with a range of mutations that the more drastic a hydrophobicity change in the H-region causes a loss of SRP client mRNA (15). We applied the Boman Index (63), a parameter to estimate protein binding, to predict SRP interaction with each signal peptide H-region variant. The Boman Index is the sum of the solubility values for all amino acid residues in a protein sequence divided by the number of residues. In our analysis, Boman Index provides an overall estimate of peptide binding to SRP. When the Boman Index value is high, the protein has a high binding potential. Based on our previous experimental analyses of disease-causing mutations in the signal peptides (15), we found that changes in the Boman index of these secretory and membrane proteins correlate well with the observed changes in the mRNA expression (Figure 3C). Therefore, changes in the Boman index score induced by signal peptide variants can be used to predict decay of the mutant protein mRNAs. As a result, we identified 8539 missense variants, or 23% of total H-region missense variants which substantially decreased both the hydrophobicity and the signal peptide-SRP interaction and classified them as PPVs due to their propensity to activate RAPP (Figure 3D, Supplementary File S3 PPV Class).

### Variants at or near the signal peptidase cleavage site leading to preprotein processing failure

**Figure 4.**
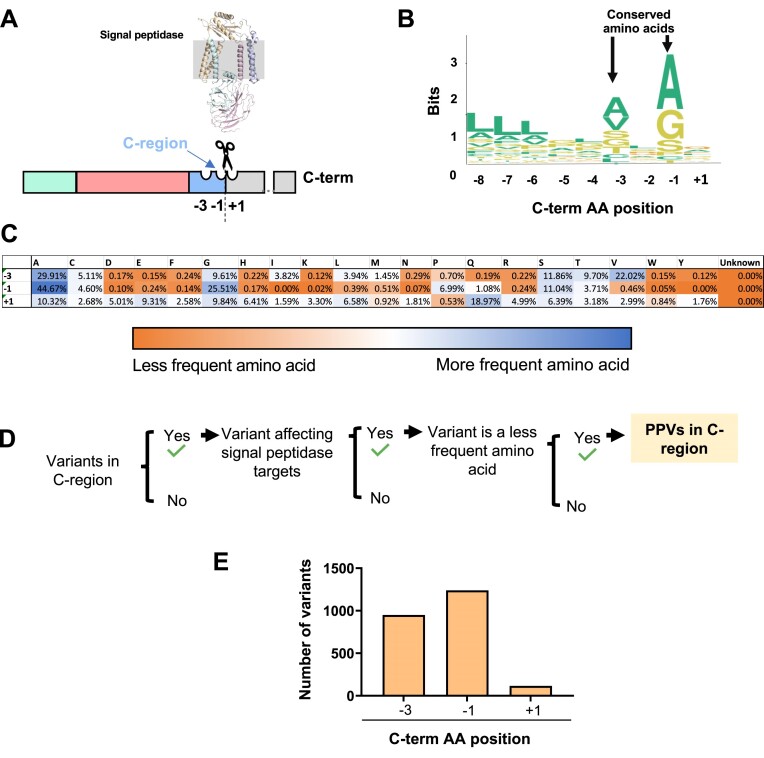
The incorporation of less frequent amino acids in signal peptidase cleavage region potentially leads to signal peptide processing failure and disease. (**A**) Schematic representation of the signal peptidase targeting the signal peptide C-region. The structure of signal peptidase complex is visualized within the ER lipid bilayer. (**B**) Protein sequence logo plot. Amino acid postions respectively to the signal peptide cleavage site are shown on the X-axis. The height of amino acid symbols within the stack indicates the relative frequency of each amino per position measure in bits (Y-axis). (**C**) Table of the relative frequency of each amino acid per signal peptide position. Less to more frequent amino acids are indicated with blue and orange, respectively. Middle values are indicated with white. (**D**) Summary of the strategy used for identifying PPVs that potentially affect the signal peptide cleavage by signal peptidase. (**E**) Distribution of PPVs per signal peptide amino acid position.

We identified 16 126 missense variants in the C-terminal region and +1 position upstream of the cleavage site (Figure 4, Supplementary File S3 PPV Class). While variants in the signal peptide H-region can impair recognition by SRP, variants in the signal peptide C-region can affect the cleavage of the signal peptide that is required to generate mature proteins (Figure 1C). The signal peptide C-region is characterized by the (–3, –1) rule which specifies restrictions for the amino acids at –3 and –1 positions near the cleavage site (–1 is the amino acid on the N-terminal side of the cleavage site while +1 is the amino acid on the C-terminal side of the cleavage site) (22). In our analysis, positions –3 and –1 are notably more conserved (e.g. alanine consists of >25% of residues in –1 and –3) than position +1 (Figure 4B), which agrees with the ‘–3, –1’ rule that small, neutral amino acids are predominant in these positions (Figure 4C). We used residue abundance at each position in the C-region in the wild type sequence to predict missense variants that lead to impaired signal peptide cleavage (Figure 4D). For example, tyrosine is a rare (<0.5%) amino acid in the –3, –1 and +1 positions (Figure 4C); thus, a missense mutation to tyrosine would be considered a PPV in any of those positions. As a result, 2267 missense variants (14% of all missense variants detected in the C-region) were retrieved and classified as PPVs. We show their distribution among amino acid positions in the C-region in Figure 4E.

To validate PPVs in the C-region, we compared our prediction with the data available in the ClinVar repository. ClinVar archives the relationships between human genetic variation and phenotypic expression with references and automatically archives any variant reported in other databases. Using this tool, we observed that 16 out of the 19 C-region variants (84%) in the ClinVar repository affect the –3 and –1 positions, and we identified 12 of them (75%) as PPVs by our strategy (Supplementary file S4).

### Signal peptide N-region variants that likely affect protein translocation through the SEC61 translocon

**Figure 5.**
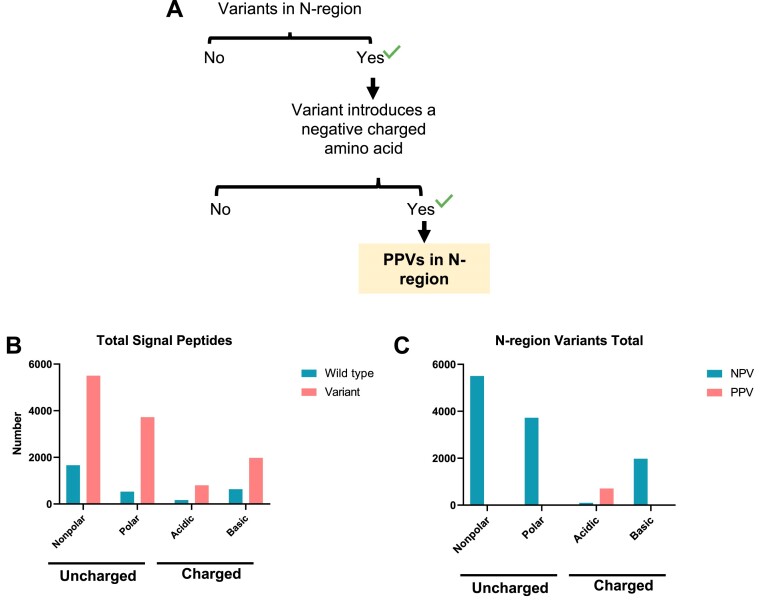
Variants introducing negatively charged amino acids in the signal peptide N-region are potentially pathogenic. The presence of positively charged amino acids is a main trait of signal peptide N-region. The N-terminal positive charge is required to orientate the nascent polypeptide across SEC61 translocon. The incorporation of negatively charge amino acids potentially impair signal peptide orientation. (**A**) Strategy for selecting PPVs in the N-region. (**B**) Bar chart summarizing the non-polar, polar, negative and positive amino acids detected in all wildtype and mutated signal peptides. (**C**) Bar chart summarizing the total variant amino acid distribution in non-pathogenic and pathogenic variants.

While the roles of the signal peptide H- and C-regions in SRP recognition and signal peptide cleavage are relatively well established, the function of the N-region is not well understood. Residues in the N-region affect the secretion efficiency of bacterial proteins (76), and the presence of positively charged amino acids (lysine and arginine) in this region is important for correct orientation of preproteins in the SEC61 translocon in eukaryotes (77). The positive charge of the N-region is also particularly important for the efficient translocation of small secretory proteins in humans (28). Thus, missense variants affecting the positive charge of the N-region likely result in decreased translocation efficiency and contribute to disease (Figures 1D, 5A). The association of N-region variants with clinical disease is less evident than for the H-region or the C-region, and most of the negatively charged N-region variants in the ClinVar repository annotated ‘uncertain significance’ due to limited data available. We identified in wild-type N-region sequences that acidic residues are not common (Figure 5B). Further, our analysis revealed 12 003 missense variants in the N-region, and 705 (6%) of these introduced negatively charged amino acids and were identified as PPVs (Figure 5C). Although the clinical data for signal peptide N-domain variants are still minimal, our evaluation provides a concept for contribution of mutations in this region to human diseases.

### Computational modeling of SRP and signal peptide interactions

**Figure 6.**
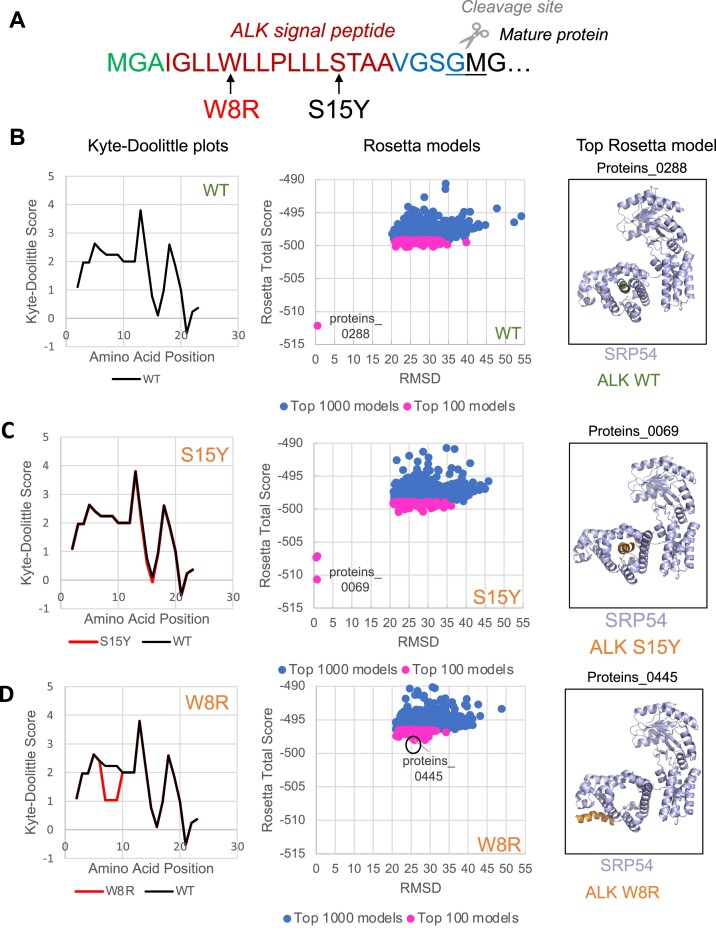
*in silico* molecular modeling of SRP54 interactions with signal peptides. (**A**) Positions of mutations W8R (PPV, marked in red) and S15Y (NPV, marked in black) in the signal peptide of ALK receptor tyrosine kinase. Signal peptide was predicted by SignalP6.0. (B–D) Modeling of the wild-type ALK signal peptide (**B**) and the mutants, S15Y (**C**), W8R (**D**). The hydrophobicity of each signal peptide was determined using the Kyte-Doolittle scale and shown in the left panels. Signal peptide mutants (red line) are compared to the WT ALK signal peptide (black line) to determine the predicted change in hydrophobicity. The top 100 (pink) and top 1000 (blue) models of the corresponding signal peptides and SRP are created using ROSIE (Rosetta Online Serve that Includes Everyone) and their distribution is shown in the central panels. The top models, which are most minimized, are labeled. These top models were selected and visualized by the use of PyMol and shown in the right panels. Wild-type signal peptide (WT) is shown in green, mutated signal peptides are in orange, and M-domain of SRP54, a subunit of SRP, is shown in light blue.

To test how mutations in signal peptides potentially affect interaction with SRP and verify our prediction of the pathogenic variants and their possible molecular mechanisms, we created *in-silico* models to show the interaction of wild-type and mutated signal peptides with SRP54, a subunit of SRP. We selected ALK protein (ALK receptor tyrosine kinase) for this analysis. ALK is a representative membrane protein, and as we found, it is one of the proteins containing multiple mutations in the signal peptide (Supplementary Files S3 and S5). Our model used amino acids 1–20 of the ALK signal peptide; this region covers the entire N- and H-regions and a part of the C-region as determined by SignalP6.0 (Figure 6A). We chose two different mutations: W8R, which is predicted to affect hydrophobicity dramatically and is designated a PPV according to our algorithm, and S15Y, which does not decrease hydrophobicity and is not a PPV (Figure 6). We used ROSIE (Rosetta Online Server that Includes Everyone) (66,67,78) to determine the best protein folding model and find whether the H-domain of ALK signal peptides will dock into the SRP54 M-domain hydrophobic pocket. We graphed the distribution of the top 100, and 1000 models in Rosetta Total Score and root-mean-square-deviation (RMSD) coordinates in Figure 6B–D, central panels. Lower RMSD values indicate more similar structures to the reference, and we indicated the most minimized, and therefore more reliable, models. Comparing models of SRP54 with WT and S15Y signal peptides, we observed that both signal peptides (WT and S15Y) are predicted to be in the hydrophobic pocket of SRP54 (bottom left dots in the graphs, Figure 6B, C, central panels). In contrast, the ALK W8R mutant does not show models in the bottom left of the graph and therefore does not have a structure that matches the reference inside the SRP54 hydrophobic pocket (Figure 6D, central panel). We then used PyMol (68) to visualize the indicated top Rosetta models to determine where the signal peptides are docking with SRP54 (Figure 6B–D, right panels). Supplementary File S11 provides validation of SRP54 Rosetta models with different ALK signal peptides. SRP54 with ALK WT and SRP54 with ALK S15Y are valid as models, but SRP54 with ALK W8R is not valid as a model demonstrating that SRP54 and ALKW8R do not interact, and, thus, in agreement with our prediction.

Our analyses demonstrate that while wild-type and S15Y variant signal peptides successfully dock in the SRP54 hydrophobic pocket crucial for signal peptide recognition, W8R does not (Figure 6B–D, right panels). Earlier, we showed that the loss of SRP interaction with signal peptide triggers RAPP protein quality control leading to mRNA degradation. Thus, we propose that activation of the RAPP pathway and loss of the ALK expression is a molecular mechanism of pathologies associated with W8R mutation. Thus, using the *in-silico* molecular modelling, we are able to distinguish between predicted pathogenic and non-pathogenic variants. This approach may be helpful for a detailed evaluation of mutation outcomes for other proteins in wide applications.

### Validation of PPVs by experimental data extracted from the literature

To verify that our strategy can identify variants that lead to RAPP and induce mRNA decay, we searched the literature for studies that report mRNA expression for proteins with signal peptide variants. Though few studies quantitatively analyzed signal peptide mutant mRNA and protein expression, our predictions match the experimentally evaluated mRNA levels expressed in different mutant cell lines (Table 1).

**Table 1.** Predicted and observed mRNA expression levels for signal peptide variants

Gene symbol	Protein length	Variant	Amino acid modification	Wild type signal peptide	Mutant signal peptide	Prediction	Experimental evidence	Cell line	Reference
AGA	346	rs386833429	15(L/R)	MARKSNLPVLLVPF**L**LCQALVRC**|**S	MARKSNLPVLLVPF**R**LCQALVRC**|**S	mRNA decay	Decreased mRNA	HeLa	(15)
CTSK	329	rs1057517252	7(L/P)	MWGLKVLL**L**PVVSFA**|**L	MWGLKV**P**LLPVVSFA**|**L	mRNA decay	Decreased mRNA	HeLa	(15)
CTSK	329	rs1057517252	9(L/P)	MWGLKVLL**L**PVVSFA**|**L	MWGLKVLL**P**PVVSFA**|**L	mRNA decay	Decreased mRNA	HeLa	(15)
GRN	593	rs63751243	9(A/D)	MWTLVSWV**A**LTAGLVAG**|**T	MWTLVSWV**D**LTAGLVAG**|**T	mRNA decay	Decreased mRNA	HeLa	(24)
INS	110	rs121908259	6(R/H)	MALWM**R**LLPLLALLALWG PDPAAA**|**F	MALWM**H**LLPLLALLALWG PDPAAA**|**F	No mRNA decay	No change in mRNA	HEK293	(51)
INS	110	rs121908278	6(R/C)	MALWM**R**LLPLLALLALWG PDPAAA**|**F	MALWM**C**LLPLLALLALWG PDPAAA**|**F	No mRNA decay	No change in mRNA	HEK293	(51)
LHCGR	699	rs4539842	16(L/Q)	MKQRFSALQLLKLLL**L**LQ PPLPRA**|**L	MKQRFSALQLLKLLL**Q**LQ PPLPRA**|**L	mRNA decay	Decreased mRNA	HEK293T	(52)
LHCGR	699	rs917607255	10(L/P)	MKQRFSALQ**L**LKLLLLLQ PPLPRA**|**L	MKQRFSALQ**P**LKLLLLLQ PPLPRA**|**L	No mRNA decay	No change in mRNA	COS-7	(53)
NDP	133	rs104894879	13(L/R)	MRKHVLAASFSM**L**SLLVI MGDTDS**|**K	MRKHVLAASFSM**R**SLLVI MGDTDS**|**K	mRNA decay	Decreased mRNA	HeLa	(15)
POMC	267	rs779629993	15(A/G)	MPRSCCSRSGALLL**A**LLLQ ASMEVRG**|**W	MPRSCCSRSGALLL**G**LLLQ ASMEVRG**|**W	No mRNA decay	No change in mRNA	β-TC3	(54)
PTH	115	rs104894271	18(C/R)	MIPAKDMAKVMIVMLAI**C**F LTKSDG**|**K	MIPAKDMAKVMIVMLAI**R**F LTKSDG**|**K	mRNA decay	Decreased mRNA	HeLa	(15)
SERPINE1	402	rs6092	15(A/T)	MQMSPALTCLVLGL**A**LV FGEGSA**|**V	MQMSPALTCLVLGL**T**LV FGEGSA**|**V	No mRNA decay	No change in mRNA	HeLa	(15)
TGFB1	390	rs1800470	10(P/L)	MPPSGLRLL**P**LLLPLLWL LVLTPGRPAAG**|**L	MPPSGLRLL**L**LLLPLLWL LVLTPGRPAAG**|**L	No mRNA decay	No change in mRNA	HeLa	(15)
UGT1A1	533	rs111033541	15(L/R)	MAVESQGGRPLVLG**L**LLC VLGPVVSHA**|**G	MAVESQGGRPLVLG**R**LLC VLGPVVSHA**|**G	mRNA decay	Decreased mRNA	HeLa	(15)
LIPA	399	rs1051338	16(T/P)	MKMRFLGLVVCLVLW**T**LH SEGSG**|**G	MKMRFLGLVVCLVLW**P**LH SEGSG**|**G	No mRNA decay	Increased mRNA	Human monocytes (CD14+)	(55)

Variants affecting the H-region were classified by their predicted effect on the mRNA level based on the potential RAPP induction. The predicted variant effects were compared with published experimental data. All variants matched the predicted and observed results. Aspartylglucosaminidase (AGA), cathepsin K (CTSK), granulin precursor (GRN), insulin (INS), luteinizing hormone/choriogonadotropin receptor (LHCGR), lipase A (LIPA), norrin cystine knot growth factor (NDP), proopiomelanocortin (POMC), parathyroid hormone (PTH), serpin family E member 1 (SERPINE1), transforming growth factor beta 1 (TGFB1), UDP glucuronosyltransferase family 1 member A (UGT1A1). Sequences presented are signal peptides plus one amino acid, the cleavage site is marked by a red line. Changed amino acids in mutant signal peptides are in red bold font, corresponding amino acids in the wild-type signal peptides are in black bold font.

Once we classified variants as PPV, we compared our list with the information available in ClinVar. Based on clinical data, this database classifies human variants in terms of their pathogenic effect. As a result, of the 43 verified pathogenic variants that affect the signal peptide H-region, 30 (∼70%) were correctly identified as PPV by our algorithm (Supplementary file S4). Thus, we could predict most of the clinical variants modifying the signal peptide H-region.

Together, our data demonstrate that the proposed bioinformatic strategy can be used to identify human signal peptide variants which impair SRP recognition and activate protein quality control pathways leading to different human diseases.

### Predicted pathogenic variants in human diseases

**Figure 7.**
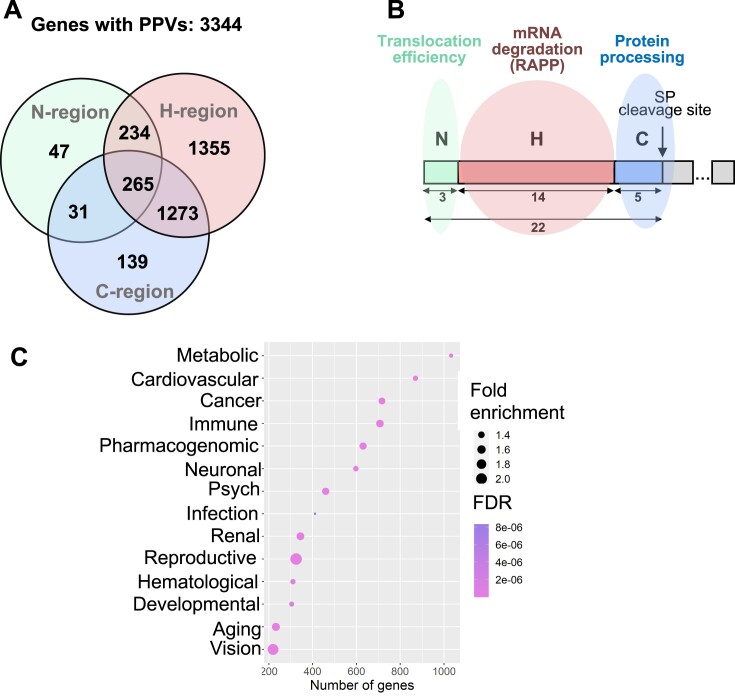
Potentially pathogenic signal peptide variants are connected with disease-associated genes and cause multiple molecular mechanisms. (**A**) Venn diagram summarizing the distribution of genes with PPVs per affected signal peptide region. (**B**) Possible molecular mechanisms of PPVs. Scheme of the typical signal peptide with marked regions is shown. Numbers represent average lengths of the signal peptide or its respected regions in amino acid residues as determined in this work. PPVs, localyzed in the signal peptide N-region, affect protein translocation efficiency through the translocon; PPVs, localyzed in the H-region, trigger mRNA degradation of the secreted/membrane protein through the RAPP pathway; and PPVs localyzed in the C-region inhibit protein processing. (**C**) Dot plot showing the number of genes per disease class. False Discovery Rate (FDR) as raw *P*-value correction. Fold enrichment obtained through comparing the background frequency of total genes annotated per disease class to the sample frequency representing the number of genes inputted that fall under the same disease class.

Using our strategy and taking into account all signal peptide regions, we identified 65 655 variants and 11 622 of them as PPVs (Supplementary file S3). These variants affect 3506 genes, and we found PPVs in different locations of the corresponding signal peptides in 3344 of these genes (162 genes of 3506 do not have any classified PPVs) (Figure 7A, Supplementary File S3). Based on our analysis and potential molecular mechanisms (Figure 1), we propose that PPVs associated with the signal peptide N-region affect signal peptide orientation and efficiency of protein translocation through the SEC61 translocon; PPVs associated with H-region induce the RAPP pathway with mRNA degradation of the secreted/membrane protein; and PPVs associated with C-region inhibit signal peptide cleavage affecting protein processing (Figure 7B). To evaluate the potential association of PPVs with particular diseases, we investigated the linkage of these genes with human disease using the Genetic Association Disease Database (https://geneticassociationdb.nih.gov/). Most genes with detected PPV are connected with metabolic and cardiovascular diseases and cancer. Reproductive and vision disease variants had a higher fold of enrichment compared to genes associated with a particular disease (Figure 7C). Remarkably, several PPVs contribute to the most severe human diseases defined in mortality (79). The identified PPVs are associated with the development of coronary heart disease (ACE, THBD, LDLR, etc.), ischemic stroke (F5, GP1BA), Alzheimer's (APOE, CD33, TREM2, SORL1), neonatal conditions(SFTPA1, SFTPA2, SFTPB, SFTPD), lung cancer (SEMA3B, WNT5B, RECK), respiratory infection (ADAM33, CCL1, CXCL1, MUC1), diabetes mellitus (INS, EGFL8, KIR3DL1), diarrheal diseases (LTF, UGT1A7, UGT1A8, UGT1A9, UGT2B7), and kidney disease (ACE, AGT, PXDN, COLEC11). The Supplementary files S6-8 summarize the distribution of genes with associated PPV per illness class and disease.

Furthermore, since PPVs may reduce protein levels and lead to loss of protein function, we also evaluated the association between decreased protein expression and associated diseases and cellular function by using Ingenuity Pathway Analysis (QIAGEN). Based on experimental data, the loss of protein function is mainly associated with increased cell death and cancer (Supplementary file S8). Together, our results demonstrate that the bioinformatics approaches applied in this study allow us to associate PPVs with particular human diseases and connect them with a molecular basis of the disorders based on the severity of the mutation and its position in the signal peptide regions.

## Discussion

The association between gene variants of protein targeting signals and human diseases remains poorly explored. In this work, we developed a bioinformatic approach to identify and classify PPVs affecting signal peptide coding sequences. We identified 65 655 missense variants in signal peptides across the human genome and predicted 11 622 of these variants as pathogenic. Our data indicates that previously reported pathogenic variants affecting signal peptides are only a tip of the iceberg – our findings significantly widen the scope of the studies on diseases associated with secretory/membrane proteins. We have highlighted that signal peptides can be impaired differently depending on their affected regions, resulting in distinct molecular mechanisms (Figure 1B, C, Figures 3–5). PPV mechanisms are summarized in Figure 7B. PPVs likely induce loss of protein expression through mRNA degradation, decreased protein translocation efficiency, protein mistargeting, or cleavage inhibition (7,24,50). The predicted pathogenic variants can be used for further analysis with future medical applications, including targeting mutations’ effects by drug development.

SRP-signal peptide interaction is an essential step in most secreted and membrane protein biogenesis. Mutations in the SRP subunits are associated with many human diseases (13). The signal peptide H-region plays the most crucial role in the process of signal peptide recognition by SRP (23). The current work identified 37 526 mutations in this region, and classified 8614 as PPVs. We also used computational modeling to simulate SRP interactions with wild-type and mutant ALK signal peptides to demonstrate how PPVs may interact with SRP. These models clearly show that the ALK signal peptide containing a charged amino acid in the H-domain cannot interact with the SRP54 subunit of SRP (Figure 6). We have previously demonstrated that mutations in the signal peptide H-region trigger the RAPP protein quality control leading to the degradation of the mutant protein mRNAs (15,24,25). In the current work, we predict that variants reducing the signal peptide hydrophobicity, and decreasing the potential signal peptide binding to SRP (Figure 3), trigger mRNA decay as a characteristic for the RAPP activation (see comparison of the prediction with published experimental data in Table 1). Thus, pathological activation of the RAPP pathway is the most likely molecular mechanism of these signal peptide variants.

However, even if a mutated secretory protein is still targeted to the ER membrane, it may be translocated inefficiently. Signal peptide hydrophobicity also determines whether signal peptides can autonomously facilitate the opening of the SEC61 translocon (‘strongly’ gating signal peptides) or if their translocation requires additional components, such as the translocon-associated protein (TRAP), SEC62 or SEC63 (80–83). Compared to strong hydrophobic signal peptides, proteins with weak hydrophobic signal peptides translocate less efficiently. It was shown recently that some proteins with weak hydrophobic transmembrane spans and signal peptides retained at the SEC61 translocon, and they need engagement of SEC63 and BiP for their release from SEC61, their translocation and folding in ER (83). Thus, some mutations that decrease the hydrophobicity of the signal peptide may result in less efficient translocation and may engage other components of the protein transport machinery to compensate the translocation defects or result in degradation of defective proteins.

In contrast, mutations that increase the hydrophobicity of the H-region are predicted to increase the interaction between SRP and the signal peptide. It was demonstrated previously that an increase in signal peptide hydrophobicity also leads an increase in pulling force, suggesting faster translocation through the SEC61 translocon (84). In fact, there appears to be both lower and upper hydrophobicity bounds for signal peptide insertion in the ER membrane. There is a hydrophobicity barrier that has to be overcome for the signal peptide or transmembrane span to be pulled through SEC61 (84–86). It was also shown that increasing the signal peptide hydrophobicity of pseudorabies virus glycoprotein gC impaired proper ER translocation (87). It was shown that highly hydrophobic signal peptides do not engage BiP during translocation, thus, these proteins may indicate a propensity to misfolding and aggregation in stress conditions (83). Only 11% of H-region variants we analyzed moderately or significantly increased hydrophobicity (Supplementary File S3), and only seven variants were found in ClinVar. Thus, the pathology of mutations increasing signal peptide hydrophobicity is still questionable. However, if some of these variants are associated with a disease, the molecular mechanism is likely due to defects in their translocation and folding, but not related to inefficient recognition by SRP inducing RAPP.

The variants introducing changes to the signal peptide in the C-region, which modify the specific amino acids recognized by signal peptidase (position –3, –1 and +1 with respect to the cleavage site), are also predicted as pathogenic by decreasing the likelihood of correct signal peptide cleavage. We show in the Figure 4B, C, that there are clear preferences for specific amino acids in the positions –3 and –1, while the appearance of the others was scarce. The +1 position was less conservative than the –1 and –3 positions, with only methionine, proline, and tryptophan being relatively rare. Variants incorporating less conserved amino acids are potentially more likely to confer disease risk and were considered PPVs (88–90) (Figure 4C, D). Most PPVs detected in the C-region affect the –1 position (Figure 4E). The –1 position corresponds to the more conserved position in the signal peptide C-region of wild-type sequences (Figure 4B). Most pathogenic variants affecting the C-region in the ClinVar repository are at the –1 position (Supplementary file S4). More information regarding the distribution of amino acids in the C-region, particularly between algorithms and the distribution of PPVs, is presented in Supplementary Files S2 and S3. These results support the idea that human missense variants affecting the amino acids targeted by signal peptidase and particularly in the –1 position promote the retention of membrane/secreted pre-proteins at the ER membrane and, induce protein loss of function and increase the risk of human diseases (26,27). Thus, the molecular mechanism of the pathogenic signal peptide variants near the cleavage site is likely associated with defects in the protein processing and, consequently, protein degradation and the loss of function.

The signal peptide N-region is less conserved than the C-region. In bacteria, this region determines the efficiency of protein secretion and possible interaction with membrane lipids. Mutations in this region do not dramatically interfere with SRP recognition, although they slightly modulate that process in mammals. Few experimental findings suggest that the correct orientation of signal peptide across the SEC61 translocon requires positively charged amino acids distributed in the N-terminal region. Small proteins are susceptible to losing the positive charge in the signal peptide N-region. As a result, the newly synthesized pre-proteins are accumulated intracellularly because of impaired translocation (28). We propose that variants incorporating negatively charged amino acids in the signal peptide N-region have a higher chance of inducing impaired translocation by affecting the required signal peptide N-terminal positive charge (Figure 5). Indeed, a group of 38 small proteins, including HCRT (narcolepsy, rs1327645071), CCL2 (neural tube defects and HIV, rs898151976), CCL7 (nephrogenic systemic fibrosis and toxic black mold infections, rs1439804640), CCL8 (tenosynovitis and T-Cell non-Hodgkin Lymphoma), INSL5 (Cryptorchidism, rs751653318), IGF1 (Pituitary Gland Disease, rs3730195), GYPA (malaria susceptibility, rs371519566) and others showed gene variants affecting the N-region charge. With our classification system, it will be much easier to identify and validate experimentally whether the human missense variants disturbing the N-region charge affect the translocation of these proteins.

Our findings provide a conceptual map for establishing the molecular basis for many human diseases associated with signal peptide mutations. It can serve as a source of PPVs and their association with the molecular mechanism of diseases for a broad group of academic and clinical researchers. Moreover, our analyses can be used to identify new and currently unknown PPVs using genome sequencing data obtained after this publication. Our classification system provides an important starting point for further models that will need to include other factors such as dominant, recessive, or dominant-negative mutations, variant frequency, gene copy number, mature protein features (size and domains), presence of homologous proteins, the effect of loss of function mutations, SRP dependency, gender, and estimated impact on fundamental biological processes.

## Supplementary Material

lqad093_Supplemental_Files

## Data Availability

The analyses are presented in the Supplementary Data to the manuscript and available in the online version. Models are submitted to ModelArchive (modelarchive.org). The individual links for the models are: SRP54 with ALK WT signal peptide: modelarchive.org/doi/10.5452/ma-owxf7 SRP54 with ALK W8R signal peptide: modelarchive.org/doi/10.5452/ma-w701z SRP54 with ALK S15Y signal peptide: modelarchive.org/doi/10.5452/ma-cm6gn

## References

[1] Thul P.J. , Åkesson L., Wiking M., Mahdessian D., Geladaki A., Ait Blal H., Alm T., Asplund A., Björk L., Breckels L.M. et al. A subcellular map of the human proteome. Science. 2017; 356:eaal3321. 28495876 10.1126/science.aal3321

[2] Sommer M.S. , Schleiff E. Protein targeting and transport as a necessary consequence of increased cellular complexity. Cold Spring Harb. Perspect. Biol. 2014; 6:a016055. 25085907 10.1101/cshperspect.a016055 PMC4107987

[3] Kunze M. , Berger J. The similarity between N-terminal targeting signals for protein import into different organelles and its evolutionary relevance. Front. Physiol. 2015; 6:259. 26441678 10.3389/fphys.2015.00259 PMC4585086

[4] von Heijne G. Signal sequences. The limits of variation. J. Mol. Biol. 1985; 184:99–105. 4032478 10.1016/0022-2836(85)90046-4

[5] von Heijne G. The signal peptide. J. Membr. Biol. 1990; 115:195–201. 2197415 10.1007/BF01868635

[6] Nielsen H. , Tsirigos K.D., Brunak S., von Heijne G. A brief history of protein sorting prediction. Protein J. 2019; 38:200–216. 31119599 10.1007/s10930-019-09838-3 PMC6589146

[7] Karamyshev A.L. , Tikhonova E.B., Karamysheva Z.N. Translational control of secretory proteins in health and disease. Int. J. Mol. Sci. 2020; 21:2538. 32268488 10.3390/ijms21072538 PMC7177344

[8] Uhlén M. , Karlsson M.J., Hober A., Svensson A.S., Scheffel J., Kotol D., Zhong W., Tebani A., Strandberg L., Edfors F. et al. The human secretome. Sci. Signal. 2019; 12:eaaz0274. 31772123 10.1126/scisignal.aaz0274

[9] Lang S. , Zimmermann R. Mechanisms of ER Protein Import. Int. J. Mol. Sci. 2022; 23:5315. 35628123 10.3390/ijms23105315 PMC9141711

[10] Uhlen M. , Fagerberg L., Hallstrom B.M., Lindskog C., Oksvold P., Mardinoglu A., Sivertsson A., Kampf C., Sjostedt E., Asplund A. et al. Proteomics. Tissue-based map of the human proteome. Science. 2015; 347:1260419. 25613900 10.1126/science.1260419

[11] Zimmermann R. , Muller L., Wullich B. Protein transport into the endoplasmic reticulum: mechanisms and pathologies. Trends Mol. Med. 2006; 12:567–573. 17071140 10.1016/j.molmed.2006.10.004

[12] Hebert D.N. , Molinari M. In and out of the ER: protein folding, quality control, degradation, and related human diseases. Physiol. Rev. 2007; 87:1377–1408. 17928587 10.1152/physrev.00050.2006

[13] Kellogg M.K. , Tikhonova E.B., Karamyshev A.L. Signal recognition particle in human diseases. Front. Genet. 2022; 13:898083. 35754847 10.3389/fgene.2022.898083 PMC9214365

[14] Rane N.S. , Chakrabarti O., Feigenbaum L., Hegde R.S. Signal sequence insufficiency contributes to neurodegeneration caused by transmembrane prion protein. J. Cell Biol. 2010; 188:515–526. 20156965 10.1083/jcb.200911115 PMC2828915

[15] Tikhonova E.B. , Karamysheva Z.N., von Heijne G., Karamyshev A.L. Silencing of aberrant secretory protein expression by disease-associated mutations. J. Mol. Biol. 2019; 431:2567–2580. 31100385 10.1016/j.jmb.2019.05.011 PMC6684239

[16] Kellogg M.K. , Miller S.C., Tikhonova E.B., Karamyshev A.L. SRPassing co-translational targeting: the role of the signal recognition particle in protein targeting and mRNA protection. Int. J. Mol. Sci. 2021; 22:6284. 34208095 10.3390/ijms22126284 PMC8230904

[17] Hsieh H.H. , Shan S.O. Fidelity of cotranslational protein targeting to the endoplasmic reticulum. Int. J. Mol. Sci. 2021; 23:281. 35008707 10.3390/ijms23010281 PMC8745203

[18] Akopian D. , Shen K., Zhang X., Shan S.O. Signal recognition particle: an essential protein-targeting machine. Annu. Rev. Biochem. 2013; 82:693–721. 23414305 10.1146/annurev-biochem-072711-164732 PMC3805129

[19] Aviram N. , Schuldiner M. Targeting and translocation of proteins to the endoplasmic reticulum at a glance. J. Cell Sci. 2017; 130:4079–4085. 29246967 10.1242/jcs.204396

[20] Hegde R.S. , Keenan R.J. The mechanisms of integral membrane protein biogenesis. Nat. Rev. Mol. Cell Biol. 2022; 23:107–124. 34556847 10.1038/s41580-021-00413-2 PMC12108547

[21] Elvekrog M.M. , Walter P. Dynamics of co-translational protein targeting. Curr. Opin. Chem. Biol. 2015; 29:79–86. 26517565 10.1016/j.cbpa.2015.09.016 PMC4684440

[22] von Heijne G. Patterns of amino acids near signal-sequence cleavage sites. Eur. J. Biochem. 1983; 133:17–21. 6852022 10.1111/j.1432-1033.1983.tb07424.x

[23] Nilsson I. , Lara P., Hessa T., Johnson A.E., von Heijne G., Karamyshev A.L. The code for directing proteins for translocation across ER membrane: SRP cotranslationally recognizes specific features of a signal sequence. J. Mol. Biol. 2015; 427:1191–1201. 24979680 10.1016/j.jmb.2014.06.014 PMC4277940

[24] Pinarbasi E.S. , Karamyshev A.L., Tikhonova E.B., Wu I.H., Hudson H., Thomas P.J. Pathogenic signal sequence mutations in progranulin disrupt SRP interactions required for mRNA stability. Cell Rep. 2018; 23:2844–2851. 29874572 10.1016/j.celrep.2018.05.003 PMC6097231

[25] Karamyshev A.L. , Patrick A.E., Karamysheva Z.N., Griesemer D.S., Hudson H., Tjon-Kon-Sang S., Nilsson I., Otto H., Liu Q., Rospert S. et al. Inefficient SRP interaction with a nascent chain triggers a mRNA quality control pathway. Cell. 2014; 156:146–157. 24439374 10.1016/j.cell.2013.12.017 PMC3931426

[26] Park S.Y. , Ye H., Steiner D.F., Bell G.I. Mutant proinsulin proteins associated with neonatal diabetes are retained in the endoplasmic reticulum and not efficiently secreted. Biochem. Biophys. Res. Commun. 2010; 391:1449–1454. 20034470 10.1016/j.bbrc.2009.12.090 PMC2817945

[27] Cui J. , Chen W., Sun J., Guo H., Madley R., Xiong Y., Pan X., Wang H., Tai A.W., Weiss M.A. et al. Competitive inhibition of the endoplasmic reticulum signal peptidase by non-cleavable mutant preprotein cargos. J. Biol. Chem. 2015; 290:28131–28140. 26446786 10.1074/jbc.M115.692350 PMC4653672

[28] Guo H. , Sun J., Li X., Xiong Y., Wang H., Shu H., Zhu R., Liu Q., Huang Y., Madley R. et al. Positive charge in the n-region of the signal peptide contributes to efficient post-translational translocation of small secretory preproteins. J. Biol. Chem. 2018; 293:1899–1907. 29229776 10.1074/jbc.RA117.000922 PMC5808753

[29] Guo H. , Xiong Y., Witkowski P., Cui J., Wang L.J., Sun J., Lara-Lemus R., Haataja L., Hutchison K., Shan S.O. et al. Inefficient translocation of preproinsulin contributes to pancreatic beta cell failure and late-onset diabetes. J. Biol. Chem. 2014; 289:16290–16302. 24770419 10.1074/jbc.M114.562355 PMC4047398

[30] Karamyshev A.L. , Karamysheva Z.N. Lost in translation: ribosome-associated mRNA and protein quality controls. Front. Genet. 2018; 9:431. 30337940 10.3389/fgene.2018.00431 PMC6180196

[31] Wang L. , Ye Y. Clearing traffic jams during protein translocation across membranes. Front. Cell Dev. Biol. 2020; 8:610689. 33490075 10.3389/fcell.2020.610689 PMC7820333

[32] Sun Z. , Brodsky J.L. Protein quality control in the secretory pathway. J. Cell Biol. 2019; 218:3171–3187. 31537714 10.1083/jcb.201906047 PMC6781448

[33] Tikhonova E.B. , Gutierrez Guarnizo S.A., Kellogg M.K., Karamyshev A., Dozmorov I.M., Karamysheva Z.N., Karamyshev A.L. Defective human SRP induces protein quality control and triggers stress response. J. Mol. Biol. 2022; 434:167832. 36210597 10.1016/j.jmb.2022.167832 PMC10024925

[34] Karamysheva Z.N. , Karamyshev A.L. Aberrant protein targeting activates quality control on the ribosome. Front. Cell Dev. Biol. 2023; 11:1198184. 37346176 10.3389/fcell.2023.1198184 PMC10279951

[35] Buchberger A. , Bukau B., Sommer T. Protein quality control in the cytosol and the endoplasmic reticulum: brothers in arms. Mol. Cell. 2010; 40:238–252. 20965419 10.1016/j.molcel.2010.10.001

[36] Volpi V.G. , Touvier T., D’Antonio M Endoplasmic reticulum protein quality control failure in myelin disorders. 2017; 9:162. 10.3389/fnmol.2016.00162 PMC5209374 28101003

[37] Tsai Y.C. , Weissman A.M. The unfolded protein response, degradation from endoplasmic reticulum and cancer. Genes Cancer. 2010; 1:764–778. 21331300 10.1177/1947601910383011 PMC3039444

[38] Phillips B.P. , Gomez-Navarro N., Miller E.A. Protein quality control in the endoplasmic reticulum. Curr. Opin. Cell Biol. 2020; 65:96–102. 32408120 10.1016/j.ceb.2020.04.002 PMC7588826

[39] Vembar S.S. , Brodsky J.L. One step at a time: endoplasmic reticulum-associated degradation. Nat. Rev. Mol. Cell Biol. 2008; 9:944–957. 19002207 10.1038/nrm2546 PMC2654601

[40] Abu-Safieh L. , Alrashed M., Anazi S., Alkuraya H., Khan A.O., Al-Owain M., Al-Zahrani J., Al-Abdi L., Hashem M., Al-Tarimi S. et al. Autozygome-guided exome sequencing in retinal dystrophy patients reveals pathogenetic mutations and novel candidate disease genes. Genome Res. 2013; 23:236–247. 23105016 10.1101/gr.144105.112 PMC3561865

[41] Sunthornthepvarakul T. , Churesigaew S., Ngowngarmratana S. A novel mutation of the signal peptide of the preproparathyroid hormone gene associated with autosomal recessive familial isolated hypoparathyroidism. J. Clin. Endocrinol. Metab. 1999; 84:3792–3796. 10523031 10.1210/jcem.84.10.6070

[42] Baker M. , Mackenzie I.R., Pickering-Brown S.M., Gass J., Rademakers R., Lindholm C., Snowden J., Adamson J., Sadovnick A.D., Rollinson S. et al. Mutations in progranulin cause tau-negative frontotemporal dementia linked to chromosome 17. Nature. 2006; 442:916–919. 16862116 10.1038/nature05016

[43] Baumer A. , Belli S., Trüeb R.M., Schinzel A. An autosomal dominant form of hereditary hypotrichosis simplex maps to 18p11.32-p11.23 in an Italian family. Eur. J. Hum. Genet. 2000; 8:443–448. 10878665 10.1038/sj.ejhg.5200506

[44] Machado R.D. , Southgate L., Eichstaedt C.A., Aldred M.A., Austin E.D., Best D.H., Chung W.K., Benjamin N., Elliott C.G., Eyries M. et al. Pulmonary arterial hypertension: a current perspective on established and emerging molecular genetic defects. Hum. Mutat. 2015; 36:1113–1127. 26387786 10.1002/humu.22904 PMC4822159

[45] Chou Y.H. , Pollak M.R., Brandi M.L., Toss G., Arnqvist H., Atkinson A.B., Papapoulos S.E., Marx S., Brown E.M., Seidman J.G. et al. Mutations in the human Ca(2+)-sensing-receptor gene that cause familial hypocalciuric hypercalcemia. Am. J. Hum. Genet. 1995; 56:1075–1079. 7726161 PMC1801464

[46] Malmgren B. , Lindskog S., Elgadi A., Norgren S. Clinical, histopathologic, and genetic investigation in two large families with dentinogenesis imperfecta type II. Hum. Genet. 2004; 114:491–498. 14758537 10.1007/s00439-004-1084-z

[47] Fischer J. , Bouadjar B., Heilig R., Huber M., Lefèvre C., Jobard F., Macari F., Bakija-Konsuo A., Ait-Belkacem F., Weissenbach J. et al. Mutations in the gene encoding SLURP-1 in Mal de Meleda. Hum. Mol. Genet. 2001; 10:875–880. 11285253 10.1093/hmg/10.8.875

[48] Dickinson J.L. , Sale M.M., Passmore A., FitzGerald L.M., Wheatley C.M., Burdon K.P., Craig J.E., Tengtrisorn S., Carden S.M., Maclean H. et al. Mutations in the NDP gene: contribution to Norrie disease, familial exudative vitreoretinopathy and retinopathy of prematurity. Clin. Experiment. Ophthalmol. 2006; 34:682–688. 16970763 10.1111/j.1442-9071.2006.01314.x

[49] Karamysheva Z.N. , Tikhonova E.B., Karamyshev A.L. Granulin in frontotemporal lobar degeneration: molecular mechanisms of the disease. Front. Neurosci. 2019; 13:395. 31105517 10.3389/fnins.2019.00395 PMC6494926

[50] Jarjanazi H. , Savas S., Pabalan N., Dennis J.W., Ozcelik H. Biological implications of SNPs in signal peptide domains of human proteins. Proteins. 2008; 70:394–403. 17680692 10.1002/prot.21548

[51] Meur G. , Simon A., Harun N., Virally M., Dechaume A., Bonnefond A., Fetita S., Tarasov A.I., Guillausseau P.J., Boesgaard T.W. et al. Insulin gene mutations resulting in early-onset diabetes: marked differences in clinical presentation, metabolic status, and pathogenic effect through endoplasmic reticulum retention. Diabetes. 2010; 59:653–661. 20007936 10.2337/db09-1091 PMC2828668

[52] Potorac I. , Trehan A., Szymanska K., Fudvoye J., Thiry A., Huhtaniemi I., Daly A.F., Beckers A., Parent A.S., Rivero-Muller A. Compound heterozygous mutations in the luteinizing hormone receptor signal peptide causing 46,XY disorder of sex development. Eur. J. Endocrinol. 2019; 181:K11–K20. 31167162 10.1530/EJE-19-0170

[53] Vezzoli V. , Duminuco P., Vottero A., Kleinau G., Schulein R., Minari R., Bassi I., Bernasconi S., Persani L., Bonomi M. A new variant in signal peptide of the human luteinizing hormone receptor (LHCGR) affects receptor biogenesis causing leydig cell hypoplasia. Hum. Mol. Genet. 2015; 24:6003–6012. 26246498 10.1093/hmg/ddv313

[54] Mencarelli M. , Zulian A., Cancello R., Alberti L., Gilardini L., Di Blasio A.M., Invitti C. A novel missense mutation in the signal peptide of the human POMC gene: a possible additional link between early-onset type 2 diabetes and obesity. Eur. J. Hum. Genet. 2012; 20:1290–1294. 22643178 10.1038/ejhg.2012.103 PMC3499745

[55] Evans T.D. , Zhang X., Clark R.E., Alisio A., Song E., Zhang H., Reilly M.P., Stitziel N.O., Razani B. Functional characterization of LIPA (lysosomal acid Lipase) variants associated with coronary artery disease. Arterioscler. Thromb. Vasc. Biol. 2019; 39:2480–2491. 31645127 10.1161/ATVBAHA.119.313443 PMC7050600

[56] UniProt Consortium UniProt: the universal protein knowledgebase in 2021. Nucleic Acids Res. 2020; 49:D480–D489. 10.1093/nar/gkaa1100 PMC7778908 33237286

[57] Durinck S. , Spellman P.T., Birney E., Huber W. Mapping identifiers for the integration of genomic datasets with the R/Bioconductor package biomaRt. Nat. Protoc. 2009; 4:1184–1191. 19617889 10.1038/nprot.2009.97 PMC3159387

[58] Teufel F. , Almagro Armenteros J.J., Johansen A.R., Gíslason M.H., Pihl S.I., Tsirigos K.D., Winther O., Brunak S., von Heijne G., Nielsen H. SignalP 6.0 predicts all five types of signal peptides using protein language models. Nat. Biotechnol. 2022; 40:1023–1025. 34980915 10.1038/s41587-021-01156-3 PMC9287161

[59] Smigielski E.M. , Sirotkin K., Ward M., Sherry S.T. dbSNP: a database of single nucleotide polymorphisms. Nucleic Acids Res. 2000; 28:352–355. 10592272 10.1093/nar/28.1.352 PMC102496

[60] Cingolani P. , Platts A., Wang le L., Coon M., Nguyen T., Wang L., Land S.J., Lu X., Ruden D.M. A program for annotating and predicting the effects of single nucleotide polymorphisms, SnpEff: sNPs in the genome of *Drosophila melanogaster* strain w1118; iso-2; iso-3. Fly. 2012; 6:80–92. 22728672 10.4161/fly.19695 PMC3679285

[61] McLaren W. , Gil L., Hunt S.E., Riat H.S., Ritchie G.R.S., Thormann A., Flicek P., Cunningham F. The ensembl variant effect predictor. Genome Biol. 2016; 17:122. 27268795 10.1186/s13059-016-0974-4 PMC4893825

[62] Kyte J. , Doolittle R.F. A simple method for displaying the hydropathic character of a protein. J. Mol. Biol. 1982; 157:105–132. 7108955 10.1016/0022-2836(82)90515-0

[63] Boman H.G. Antibacterial peptides: basic facts and emerging concepts. J. Intern. Med. 2003; 254:197–215. 12930229 10.1046/j.1365-2796.2003.01228.x

[64] Varadi M. , Anyango S., Deshpande M., Nair S., Natassia C., Yordanova G., Yuan D., Stroe O., Wood G., Laydon A. et al. AlphaFold Protein Structure Database: massively expanding the structural coverage of protein-sequence space with high-accuracy models. Nucleic Acids Res. 2022; 50:D439–D444. 34791371 10.1093/nar/gkab1061 PMC8728224

[65] Jumper J. , Evans R., Pritzel A., Green T., Figurnov M., Ronneberger O., Tunyasuvunakool K., Bates R., Žídek A., Potapenko A. et al. Highly accurate protein structure prediction with AlphaFold. Nature. 2021; 596:583–589. 34265844 10.1038/s41586-021-03819-2 PMC8371605

[66] Lyskov S. , Gray J.J. The RosettaDock server for local protein–protein docking. Nucleic Acids Res. 2008; 36:W233–W238. 18442991 10.1093/nar/gkn216 PMC2447798

[67] Chaudhury S. , Berrondo M., Weitzner B.D., Muthu P., Bergman H., Gray J.J. Benchmarking and analysis of protein docking performance in Rosetta v3.2. PLoS One. 2011; 6:e22477. 21829626 10.1371/journal.pone.0022477 PMC3149062

[68] The PyMOL Molecular Graphics System, Version 2.5. Schrödinger, LLC.

[69] Halic M. , Becker T., Pool M.R., Spahn C.M., Grassucci R.A., Frank J., Beckmann R. Structure of the signal recognition particle interacting with the elongation-arrested ribosome. Nature. 2004; 427:808–814. 14985753 10.1038/nature02342

[70] Gao Y. , Zhang Q., Lang Y., Liu Y., Dong X., Chen Z., Tian W., Tang J., Wu W., Tong Y. et al. Human apo-SRP72 and SRP68/72 complex structures reveal the molecular basis of protein translocation. J. Mol. Cell Biol. 2017; 9:220–230. 28369529 10.1093/jmcb/mjx010 PMC5907831

[71] Grotwinkel J.T. , Wild K., Segnitz B., Sinning I. SRP RNA remodeling by SRP68 explains its role in protein translocation. Science. 2014; 344:101–104. 24700861 10.1126/science.1249094

[72] Kuglstatter A. , Oubridge C., Nagai K. Induced structural changes of 7SL RNA during the assembly of human signal recognition particle. Nat. Struct. Biol. 2002; 9:740–744. 12244299 10.1038/nsb843

[73] Liaci A.M. , Steigenberger B., Telles de Souza P.C., Tamara S., Grollers-Mulderij M., Ogrissek P., Marrink S.J., Scheltema R.A., Forster F. Structure of the human signal peptidase complex reveals the determinants for signal peptide cleavage. Mol. Cell. 2021; 81:3934–3948. 34388369 10.1016/j.molcel.2021.07.031

[74] Thomas P.D. , Ebert D., Muruganujan A., Mushayahama T., Albou L.P., Mi H. PANTHER: making genome-scale phylogenetics accessible to all. Protein Sci. 2022; 31:8–22. 34717010 10.1002/pro.4218 PMC8740835

[75] Sherry S.T. , Ward M.H., Kholodov M., Baker J., Phan L., Smigielski E.M., Sirotkin K. dbSNP: the NCBI database of genetic variation. Nucleic Acids Res. 2001; 29:308–311. 11125122 10.1093/nar/29.1.308 PMC29783

[76] Nesmeyanova M.A. , Karamyshev A.L., Karamysheva Z.N., Kalinin A.E., Ksenzenko V.N., Kajava A.V. Positively charged lysine at the N-terminus of the signal peptide of the Escherichia coli alkaline phosphatase provides the secretion efficiency and is involved in the interaction with anionic phospholipids. FEBS Lett. 1997; 403:203–207. 9042967 10.1016/s0014-5793(97)00052-5

[77] Goder V. , Junne T., Spiess M. Sec61p Contributes to Signal Sequence Orientation According to the Positive-Inside Rule. Mol. Biol. Cell. 2003; 15:1470–1478. 14668483 10.1091/mbc.E03-08-0599 PMC363169

[78] Lyskov S. , Chou F.C., Conchúir S., Der B.S., Drew K., Kuroda D., Xu J., Weitzner B.D., Renfrew P.D., Sripakdeevong P. et al. Serverification of molecular modeling applications: the Rosetta Online Server that Includes Everyone (ROSIE). PLoS One. 2013; 8:e63906. 23717507 10.1371/journal.pone.0063906 PMC3661552

[79] World Health Organization The Top 10 Causes of Death. 2020; World Health Organization.

[80] Kriegler T. , Kiburg G., Hessa T. Translocon-Associated Protein Complex (TRAP) is crucial for co-translational translocation of pre-proinsulin. J. Mol. Biol. 2020; 432:166694. 33137310 10.1016/j.jmb.2020.10.028

[81] Jadhav B. , McKenna M., Johnson N., High S., Sinning I., Pool M.R. Mammalian SRP receptor switches the Sec61 translocase from Sec62 to SRP-dependent translocation. Nat. Commun. 2015; 6:10133. 26634806 10.1038/ncomms10133 PMC4686813

[82] Hassdenteufel S. , Johnson N., Paton A.W., Paton J.C., High S., Zimmermann R. Chaperone-mediated Sec61 channel gating during ER import of small precursor proteins overcomes Sec61 inhibitor-reinforced energy barrier. Cell Rep. 2018; 23:1373–1386. 29719251 10.1016/j.celrep.2018.03.122 PMC5946456

[83] Sun S. , Li X., Mariappan M. Signal sequences encode information for protein folding in the endoplasmic reticulum. J. Cell Biol. 2023; 222:e202203070. 36459117 10.1083/jcb.202203070 PMC9723807

[84] Kriegler T. , Magoulopoulou A., Amate Marchal R., Hessa T. Measuring endoplasmic reticulum signal sequences translocation efficiency using the Xbp1 arrest peptide. Cell Chem Biol. 2018; 25:880–890. 29754956 10.1016/j.chembiol.2018.04.006

[85] Hessa T. , Kim H., Bihlmaier K., Lundin C., Boekel J., Andersson H., Nilsson I., White S.H., von Heijne G. Recognition of transmembrane helices by the endoplasmic reticulum translocon. Nature. 2005; 433:377–381. 15674282 10.1038/nature03216

[86] Hessa T. , Meindl-Beinker N.M., Bernsel A., Kim H., Sato Y., Lerch-Bader M., Nilsson I., White S.H., von Heijne G. Molecular code for transmembrane-helix recognition by the Sec61 translocon. Nature. 2007; 450:1026–1030. 18075582 10.1038/nature06387

[87] Tomilo M. , Wilkinson K.S., Ryan P. Can a signal sequence become too hydrophobic?. J. Biol. Chem. 1994; 269:32016–32021. 7989378

[88] Pérez-Palma E. , May P., Iqbal S., Niestroj L.M., Du J., Heyne H.O., Castrillon J.A., O’Donnell-Luria A., Nürnberg P., Palotie A. et al. Identification of pathogenic variant enriched regions across genes and gene families. Genome Res. 2020; 30:62–71. 31871067 10.1101/gr.252601.119 PMC6961572

[89] Miller M.P. , Kumar S. Understanding human disease mutations through the use of interspecific genetic variation. Hum. Mol. Genet. 2001; 10:2319–2328. 11689479 10.1093/hmg/10.21.2319

[90] Vockley J.G. , Goodman B.K., Tabor D.E., Kern R.M., Jenkinson C.P., Grody W.W., Cederbaum S.D. Loss of function mutations in conserved regions of the human arginase I gene. Biochem. Mol. Med. 1996; 59:44–51. 8902193 10.1006/bmme.1996.0063

