# Supplementary material for: Pathogenic signal peptide variants in the human genome
Source: NAR Genom Bioinform. 2023 Oct 18;5(4):lqad093. doi: 10.1093/nargab/lqad093 (PMC10583284; doi:10.1093/nargab/lqad093)
Supplement: lqad093_Supplemental_Files [file lqad093_supplemental_files.zip › Supplementary-Text-Figures-Ref-List-of-Supp-Files-230806.pdf]

SUPPLEMENTARY TEXT

Signal peptides and the distribution of variants in their regions as determined by SignalP6.0 and UniProt

We used the most advanced software, SignalP6.0 (slow mode), to detect signal peptides and their regions in secretory proteins in our analysis. When we compared the data obtained with the signal peptides determined by UniProt, we found that about 17% of signal peptides have alternative lengths affecting distribution of the H- and C-regions (Supplementary Figure S1A). To predict signal peptides, UniProt uses four algorithms, SignalP5.0, Phobius, COILS (now PCOILS), and MobiDB-lite (1-7). We would like to point out that COILS and SignalP5.0 are outdated versions. The newest versions, DeepCoil and SignalP6.0, have not yet been integrated into UniProt. Although we used SignalP6.0 exclusively in our work we also completed analysis of proteins with alternative signal peptides and their variants to detect possible PPVs in those cases. We anticipate that this analysis will be valuable for researches in the secretory protein field and beyond working with these proteins – they will have ability to detailly exam their alternative forms and compare with their experimental data. The usage of the particular algorithm may be associated with alternative positioning of the mutations in C- or H-region of the signal peptides for subset of these proteins. This may affect the prediction of the mutations' impact. In this work we analyzed all possible variants (see Supplementary Figures S1-S3 and Supplementary Files S1-S3 for details). Supplementary File S2 presents data on the amino acid frequency per signal peptide region. SP Match indicates that UniProt's signal sequence region determination and SignalP6.0's reveals identical regions. SP No Match indicates that there is a difference in signal peptide length depending on which algorithm is used. SP No Match are those genes that have multiple cleavage sites detected, and SignalP6.0 and UniProt determined different lengths of signal peptide regions. UniProt is using UniProt's signal sequence determination of the +1, -1, -3 positions. SignalP6.0 uses SignalP6.0's determination of the cleavage site. The cleavage site amino acid distribution by Von Heijne (8) is used for comparison. The +1, -1, and -3 positions using various algorithms are compared in Summary Percent. Supplementary File S3 summarizes the classified PPVs. Variants are classified as either non-pathogenic (NPVs) or pathogenic (PPVs). The distribution of PPVs and NPVs are further separated into whether their signal peptides match or not, as described in Supplementary File S2. Variants in each region are classified by their amino acid

composition into four categories: Nonpolar; polar; acidic; and basic. Further, we classify the genes by the number of variants in each region or overlapping regions, e.g. whether a gene has N-, H-, C-region variants or any of the permutations within. We also used PANTHER (Protein Analysis Through Evolutionary Relationships) (9), to determine which pathways were affected by mutations in the genes that do not have agreement between the UniProt and SignalP6.0 algorithm (Supplementary Fig. 1E and Supplementary Files 10). The information presented here may be crucial for researches working with these particular proteins, and they will be able to evaluate specific mutations and their potential outcome on the base of our data.

Supplementary References

1. Almagro Armenteros, J.J., Tsirigos, K.D., Sonderby, C.K., Petersen, T.N., Winther, O., Brunak, S., von Heijne, G. and Nielsen, H. (2019) SignalP 5.0 improves signal peptide predictions using deep neural networks. *Nature biotechnology*, **37**, 420-423.
2. Krogh, A., Larsson, B., von Heijne, G. and Sonnhammer, E.L. (2001) Predicting transmembrane protein topology with a hidden Markov model: application to complete genomes. *J Mol Biol*, **305**, 567-580.
3. Kall, L., Krogh, A. and Sonnhammer, E.L. (2004) A combined transmembrane topology and signal peptide prediction method. *J Mol Biol*, **338**, 1027-1036.
4. Zimmermann, L., Stephens, A., Nam, S.Z., Rau, D., Kubler, J., Lozajic, M., Gabler, F., Soding, J., Lupas, A.N. and Alva, V. (2018) A Completely Reimplemented MPI Bioinformatics Toolkit with a New HHpred Server at its Core. *J Mol Biol*, **430**, 2237-2243.
5. Gabler, F., Nam, S.Z., Till, S., Mirdita, M., Steinegger, M., Soding, J., Lupas, A.N. and Alva, V. (2020) Protein Sequence Analysis Using the MPI Bioinformatics Toolkit. *Curr Protoc Bioinformatics*, **72**, e108.
6. Necci, M., Piovesan, D., Dosztanyi, Z. and Tosatto, S.C.E. (2017) MobiDB-lite: fast and highly specific consensus prediction of intrinsic disorder in proteins. *Bioinformatics*, **33**, 1402-1404.
7. Sonnhammer, E.L., von Heijne, G. and Krogh, A. (1998) A hidden Markov model for predicting transmembrane helices in protein sequences. *Proc Int Conf Intell Syst Mol Biol*, **6**, 175-182.
8. von Heijne, G. (1983) Patterns of amino acids near signal-sequence cleavage sites. *Eur J Biochem*, **133**, 17-21.
9. Thomas, P.D., Ebert, D., Muruganujan, A., Mushayahama, T., Albou, L.P. and Mi, H. (2022) PANTHER: Making genome-scale phylogenetics accessible to all. *Protein Sci*, **31**, 8-22.

Supplementary Figure S1

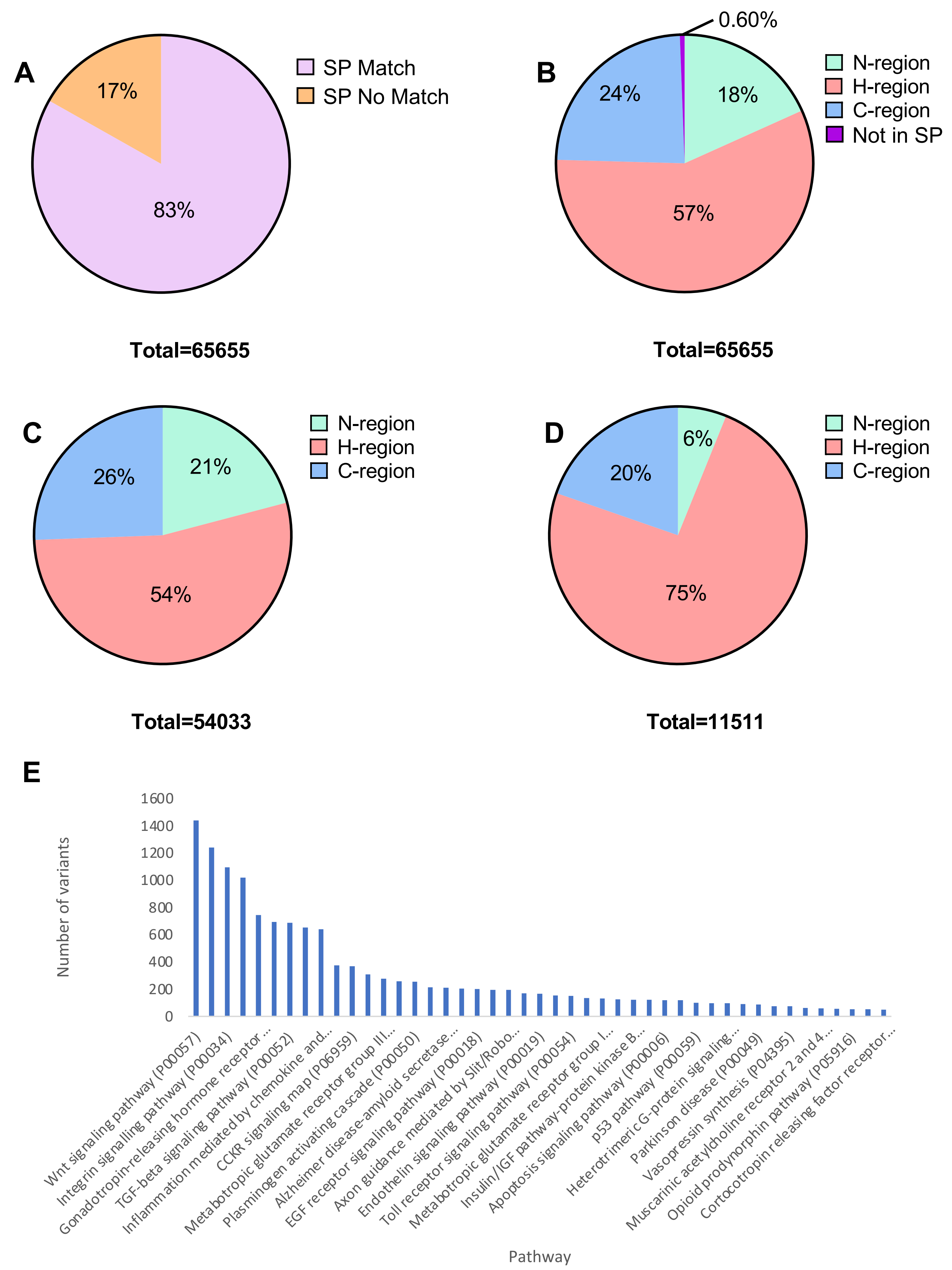

Supplementary Figure S1. Distribution of signal peptide variants using different algorithms

A) Distribution of signal peptide variants by SP Match and No Match obtained by the UniProt and SignalP6.0 algorithms. SP Match indicates that UniProt and SignalP6.0 reveal signal sequences of identical length and identical regions. SP No Match indicates that there is a difference in signal peptide length and region determination depending on which algorithm is used. **B)** Distribution of the Total Variants in SP regions based on the UniProt algorithm. The variants not in SPs (0.6%) are associated with the discrepancy of SP lengths determined by UniProt and SignalP6.0, thus some variants were outside of SPs when UniProt was used. **C)** Distribution of nonpathogenic missense variants (NPV) between different signal peptide regions using UniProt's algorithm. The signal peptide variants include those located in the position +1 because it may affect signal peptide cleavage (protein processing). **D)** Distribution of pathogenic missense variants between different signal peptide regions using UniProt's algorithm. The signal peptide variants include those located in the position +1. **E)** PANTHER pathway analysis of the genes that do not have agreement between the UniProt and SignalP6.0 algorithms. A cut-off of 50 genes was used to determine the pathways which were most affected by the algorithm difference.

Supplementary Figure S2

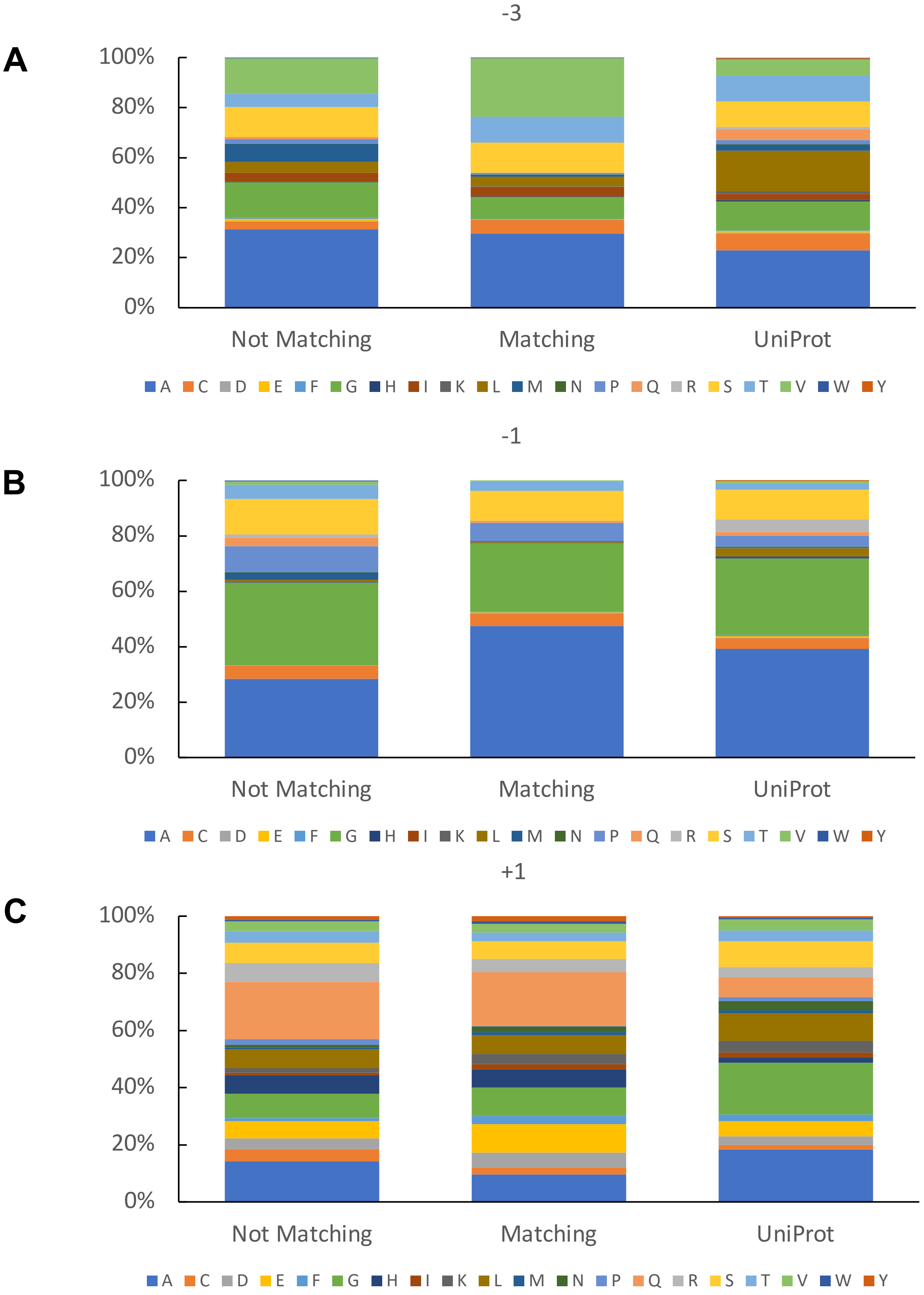

D

-3	A	C	D	E	F	G	H	I	K	L	M	N	P	Q	R	S	T	V	W	Y	Unknown
Not Matching	31.35%	3.14%	0.17%	0.83%	0.50%	14.19%	0.17%	3.63%	0.00%	4.29%	7.10%	0.17%	1.82%	0.83%	0.17%	11.88%	5.28%	14.19%	0.33%	0.00%	0.00%
Matching	29.67%	5.45%	0.17%	0.03%	0.20%	8.82%	0.23%	3.86%	0.14%	3.89%	0.48%	0.31%	0.51%	0.09%	0.23%	11.85%	10.47%	23.37%	0.11%	0.14%	0.00%
UniProt	22.96%	6.67%	0.49%	0.49%	0.25%	11.60%	0.49%	2.47%	0.99%	16.30%	2.47%	0.25%	1.48%	4.44%	0.99%	10.12%	10.37%	6.42%	0.25%	0.49%	0.00%
von Heijne 1983	28.89%	7.78%	0.00%	0.00%	0.00%	1.11%	0.00%	7.78%	0.00%	5.56%	0.00%	0.00%	0.00%	0.00%	0.00%	21.11%	8.89%	15.56%	0.00%	0.00%	3.33%
-1	A	C	D	E	F	G	H	I	K	L	M	N	P	Q	R	S	T	V	W	Y	Unknown
Not Matching	28.29%	4.88%	0.16%	0.00%	0.00%	29.76%	0.16%	0.00%	0.16%	0.81%	2.44%	0.16%	9.43%	3.09%	1.14%	12.85%	5.04%	1.30%	0.33%	0.00%	0.00%
Matching	47.52%	4.56%	0.08%	0.28%	0.17%	24.77%	0.17%	0.00%	0.00%	0.31%	0.17%	0.06%	6.57%	0.74%	0.08%	10.73%	3.48%	0.31%	0.00%	0.00%	0.00%
UniProt	39.26%	3.88%	0.16%	0.48%	0.48%	27.63%	0.32%	0.16%	0.48%	2.58%	0.16%	0.48%	4.04%	1.29%	4.52%	10.66%	2.42%	0.81%	0.00%	0.16%	0.00%
von Heijne 1983	46.67%	5.56%	0.00%	0.00%	0.00%	22.22%	0.00%	0.00%	0.00%	0.00%	0.00%	0.00%	0.00%	1.11%	0.00%	20.00%	2.22%	0.00%	0.00%	0.00%	2.22%
+1	A	C	D	E	F	G	H	I	K	L	M	N	P	Q	R	S	T	V	W	Y	Unknown
Not Matching	14.31%	4.23%	3.74%	6.02%	1.14%	8.46%	6.34%	0.98%	1.63%	6.50%	0.65%	0.98%	1.95%	20.16%	6.50%	7.15%	4.07%	3.41%	0.49%	1.30%	0.00%
Matching	9.62%	2.41%	5.24%	9.88%	2.83%	10.08%	6.43%	1.70%	3.59%	6.59%	0.96%	1.95%	0.28%	18.77%	4.73%	6.26%	3.03%	2.92%	0.91%	1.84%	0.00%
UniProt	18.26%	1.62%	3.07%	5.33%	2.26%	18.26%	1.78%	1.78%	4.04%	9.69%	0.97%	3.23%	1.45%	6.95%	3.55%	9.05%	3.72%	3.88%	0.65%	0.48%	0.00%
von Heijne 1983	6.67%	10.00%	8.89%	4.44%	6.67%	4.44%	0.00%	1.11%	7.78%	5.56%	1.11%	2.22%	0.00%	13.33%	10.00%	4.44%	3.33%	2.22%	1.11%	0.00%	6.67%

Supplementary Figure S2. Distribution of Amino Acids in the -3, -1, and +1 position near the cleavage site of the signal peptide

A) Distribution of amino acids in the -3 position. Variants that have different signal peptides between UniProt and SignalP6.0 are designated Not Matching (17% of all SPs, see Supplementary Figure S1A). Variants that have identical signal peptides between UniProt and SignalP6.0 are designated Matching (83% of all SPs). Amino acids are labeled with one letter code and the percentage of each amino acid residue in the -3 position are graphed. The percentage of all amino acids in that location add up to 100%. **B)** Distribution of amino acid residues in the -1 position using the previous schemes. **C)** Distribution of amino acid residues in the +1 position using the schemes in A. **D)** Table presents percentage distribution of amino acids in the -3, -1, and +1 positions. Distribution of amino acid residues from the original work which described the -3, -1 rule (8) included for comparison. Column "unknown" refers to unidentified amino acids in von Heijne 1983 (8). Blue represents highly conserved residues while orange represents relatively rare residues; neither conserved nor rare residues are indicated in white.

Supplementary Figure S3

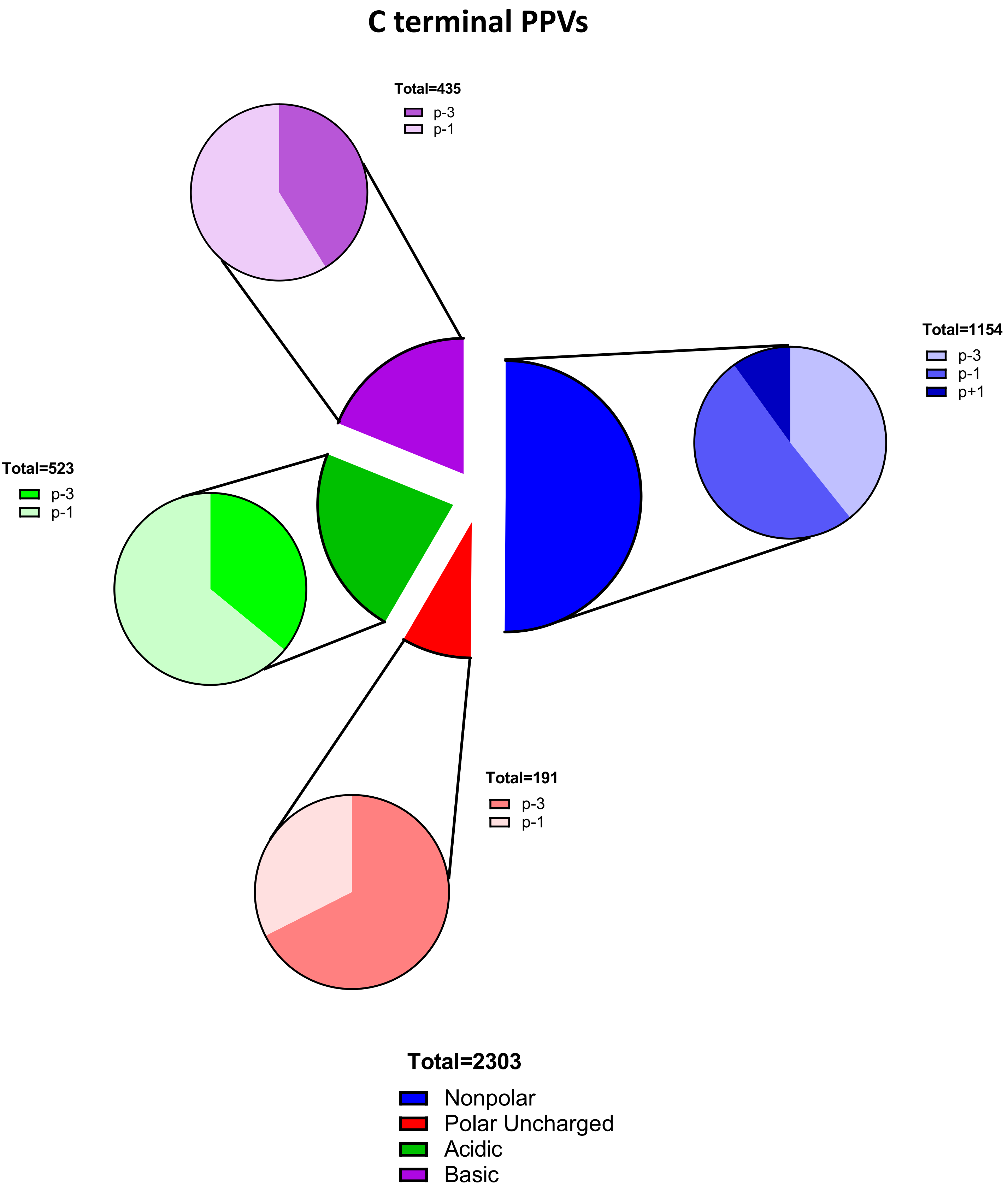

Supplementary Figure S3. **Distribution of PPVs in signal peptide C-region.**

SignalP6.0 algorithm was used to determine the SP -3, -1, and +1 positions. The pathogenic variants are broken down into the categories of nonpolar (blue), polar uncharged (red), acidic (green), and basic (purple). Basic pathogenic variants are further broken down into variants that affect -3 position (dark purple) or -1 position (light purple). Nonpolar pathogenic variants are broken down into variants that affect the -3 position (light blue), -1 position (medium blue), or +1 position (dark blue). Polar uncharged pathogenic variants are categorized as variants affecting the -3 position (medium red) or -1 position (light red). Finally, acidic variants affect the -3 position (medium green) or the -1 position (light green).

LIST OF SUPPLEMENTARY FILES

Supplementary File S1: Datasets of the signal peptide proteins recovered from the UniProtKB/Swiss-Prot and SignalP6.0 algorithm.

Supplementary File S2: Dataset summarizing the amino acid frequency per signal peptide region.

Supplementary File S3: Dataset summarizing the classified PPVs.

Supplementary File S4: Support data summarizing H- and C-region genes with PPVs and its comparison to variants reported in ClinVar.

Supplementary File S5: Dataset summarizing the classified PPVs in ALK

Supplementary File S6: Dataset of PPVs distributed per disease class in the Genetic Association Database.

Supplementary File S7: Dataset of PPVs distributed per disease and tissue in the Genetic Association Database.

Supplementary File S8: PPVs analyzed by biological processes (BioPro) using QIAGEN Ingenuity Pathway Analysis.

Supplementary File S9: R script for variant classification.

Supplementary File S10: PANTHER scores for non-matching signal peptides genes based on biological processes. The Wnt signaling pathway, cadherin and integrin signaling pathways, and the presenilin pathway, which is involved in Alzheimer's, involve proteins with differences in their signal peptide length and/or distribution of residues among the N-, H-, and C-regions.

Supplementary File S11: Validation of SRP54 Rosetta models with different ALK signal peptides.
